# Hydrogeological compartmentalization outweighs hydrochemistry in structuring microbial communities across contrasting subsurface habitats

**DOI:** 10.3389/fmicb.2026.1919949

**Published:** 2026-09-28

**Authors:** Cassandre Sara Lazar, Pierre Betti, Coralie Rolland, Jean-Sébastien Marcil

**Affiliations:** 1 Department of Biological Sciences, Université du Québec à Montréal (UQAM), Montreal, QC, Canada; 2 Derena Géosciences, Quebec City, QC, Canada

**Keywords:** *Acetobacterium*, deep biosphere, deep groundwater, environmental filtering, hydrogeological compartmentalization, methanogens, microbial community assembly, subsurface microbiology

## Abstract

Deep groundwater ecosystems are energy-limited microbial habitats in which hydrochemical conditions, hydrogeological structure, and habitat isolation jointly influence community assembly. However, the relative importance of environmental filtering and hydrogeological compartmentalization in structuring subsurface microbial communities remains poorly understood. We characterized hydrochemistry, bacterial communities, and archaeal signatures across two contrasting groundwater systems in Québec, Canada: a shallow permeable groundwater system at pointe-du-lac (PDL) and deep fractured formation waters (1,460–1,690 m) at saint-Flavien (SF). Hydrochemical analyses, bacterial and archaeal amplicon sequencing, occupancy-based approaches, and multivariate statistical analyses were used to evaluate patterns of community composition, persistence, and environmental association. The two groundwater systems differed markedly in hydrochemical composition, with PDL enriched in major ions and SF characterized by elevated trace element concentrations. Despite these differences, bacterial communities were structured primarily at the scale of individual wells rather than by groundwater system. Well identity explained a substantially larger fraction of community variation than hydrochemistry, indicating strong hydrogeological compartmentalization. PDL supported a larger recurrent bacterial component, stronger taxon–environment relationships, and repeated methanogen-associated archaeal signatures, whereas SF contained a smaller persistent community fraction and a greater contribution from localized taxa. Across both systems, *Acetobacterium* was the dominant bacterial genus and was largely represented by a single recurrent lineage distributed across multiple wells and hydrochemically distinct habitats. Recurrent taxa in PDL were enriched in anaerobic and sulfur-associated lineages, while SF was characterized by a smaller set of persistent groundwater-associated taxa. Methanogen-associated archaea were largely restricted to PDL and included both methylotrophic and hydrogenotrophic lineages. These results demonstrate that hydrogeological compartmentalization outweighs hydrochemistry in structuring microbial communities across contrasting subsurface habitats. The persistence of recurrent bacterial lineages, together with localized archaeal assemblages, suggests that deep groundwater ecosystems are shaped by the combined effects of hydrogeological compartmentalization, environmental filtering, and adaptation to long-term energy limitation.

## 1 Introduction

Groundwater systems constitute one of the largest microbial habitats on Earth and support diverse microbial communities that contribute to carbon, sulfur, nitrogen, and metal cycling (Griebler and Lueders, 2009; McMahon and Parnell, 2014; Beaver and Neufeld, 2024). Although these environments are often perceived as stable ecosystems, they encompass a broad range of physicochemical and hydrogeological conditions that can influence microbial community composition and persistence (Griebler and Lueders, 2009). Understanding the relative importance of the factors that structure groundwater microbiomes therefore remains a central challenge in subsurface microbial ecology. Among groundwater habitats, deep subsurface environments are of particular interest because they represent extreme microbial ecosystems characterized by chronic energy limitation, restricted nutrient availability, long water residence times, and varying degrees of physical isolation (Krumholz, 2000; Lever et al., 2015; Beaver and Neufeld, 2024). Microbial populations inhabiting these environments must persist under conditions that differ markedly from those encountered in surface ecosystems, making deep groundwater systems valuable natural laboratories for investigating the ecological processes that govern microbial survival, community assembly, and long-term persistence in the deep biosphere (Magnabosco et al., 2018).

Previous studies have shown that groundwater microbial communities are influenced by a combination of hydrochemical conditions, geological setting, groundwater age, depth, groundwater recharge, and host-rock lithology (Griebler and Lueders, 2009; Hug et al., 2015; Beaver and Neufeld, 2024; Wang et al., 2025). Recent studies similarly identified hydrochemical variables such as sulfate, potassium, and pH as important correlates of groundwater microbial community composition (Chen et al., 2026). Hydrochemical variables such as pH, salinity, electron acceptor availability, and dissolved metals have frequently been associated with microbial community composition because they influence resource availability and redox conditions within subsurface habitats (Griebler and Lueders, 2009; Hug et al., 2015). However, increasing evidence suggests that local hydrogeological structure may also exert strong controls on microbial assembly by limiting dispersal and generating compartmentalized habitats (Ben Maamar et al., 2015; Fillinger et al., 2019). Consequently, microbial communities can differ substantially among nearby wells despite sharing similar hydrochemical conditions (Ben Maamar et al., 2015; Fillinger et al., 2019). Understanding the relative importance of environmental filtering and hydrogeological compartmentalization remains a central challenge in groundwater microbial ecology, particularly in deep and energy-limited environments where direct observations remain scarce.

The groundwater systems investigated in this study differ markedly in their hydrogeological context. Pointe-du-Lac represents a relatively shallow groundwater environment, whereas Saint-Flavien corresponds to deep formation waters occurring at approximately 1,460 to 1,690 m depth. These contrasting settings provide an opportunity to examine how microbial communities are organized across subsurface habitats that differ substantially in depth, connectivity, and hydrogeological isolation. Comparing these systems may help identify microbial features that are shared across groundwater environments as well as those associated with contrasting modes of subsurface habitat organization. The objectives of this study were therefore to (i) characterize the hydrochemical and microbial composition of contrasting groundwater habitats, (ii) evaluate the relative contributions of hydrochemistry and hydrogeological structure to microbial community assembly, and (iii) identify bacterial and archaeal lineages across two contrasting subsurface habitats differing markedly in depth, geological setting, and hydrological connectivity.

## 2 Materials and methods

### 2.1 Study sites and sampling

Groundwater samples were collected from two subsurface natural gas storage sites located in southern Quebec, Canada: Pointe-du-Lac (PDL) and Saint-Flavien (SF) (Figure 1). Although both sites are used for underground gas storage, they differ markedly in geological setting, depth, and hydrogeological characteristics, providing an opportunity to compare microbial communities across contrasting subsurface habitats. The PDL site is located approximately 12 km southwest of Trois-Rivières. The reservoir occurs within the Pleistocene-aged Lotbinière Sand Formation at a depth of approximately 70 m below ground surface. It consists of unconsolidated sands and gravels overlain by low-permeability lacustrine clay deposits that act as a confining layer. The reservoir is characterized by high porosity (~36%) and permeability on the order of a few darcies (Lavoie et al., 2014) and is hydraulically connected to an underlying saline aquifer system (~14,000 ppm dissolved solids). Reservoir sediments include both carbonate-rich and feldspar-rich sand units. The SF site is located approximately 45 km southwest of Québec City. The reservoir occurs within fractured dolomites of the Ordovician Beauharnois Formation at depths ranging from approximately 1,460 to 1,690 m. In contrast to PDL, the formation exhibits low matrix porosity (~6% on average) and permeability (~4 mD on average, ranging from 0.1 to 70 mD), with fluid flow controlled primarily by secondary fracturing (Lavoie et al., 2011). Available pressure data indicate limited hydraulic support from surrounding aquifers, suggesting a comparatively isolated groundwater environment. Therefore, these sites represent two contrasting subsurface groundwater systems that differ substantially in depth, geological setting, reservoir lithology, reservoir properties, and inferred hydrological connectivity, making them suitable model systems for investigating the ecological factors structuring deep groundwater microbial communities.

**Figure 1:**
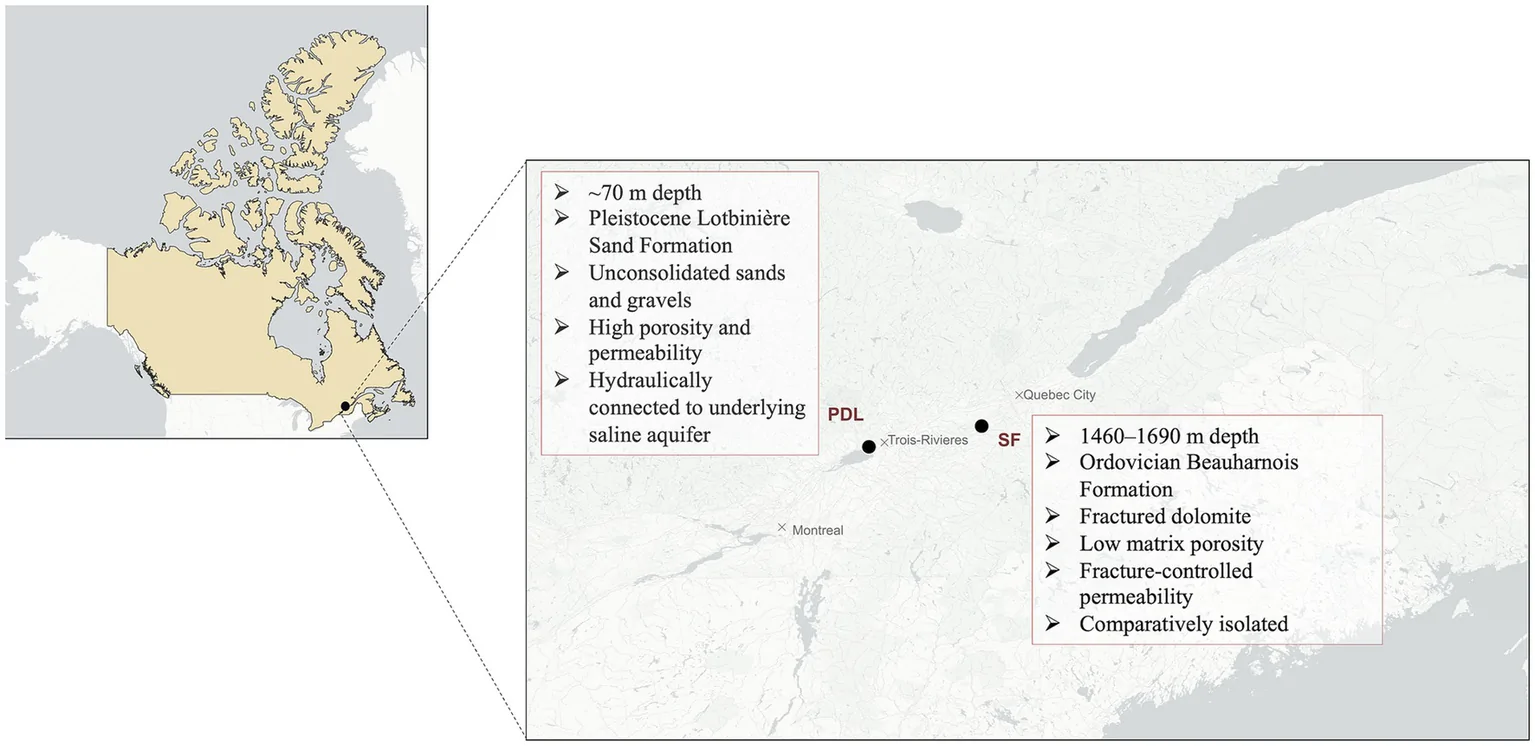
Geographic location and hydrogeological characteristics of the two groundwater study sites in southern Québec, Canada. The map shows the locations of the Pointe-du-Lac (PDL) and Saint-Flavien (SF) groundwater systems, with the inset indicating their location within Canada. Boxes summarize the principal hydrogeological characteristics distinguishing the two sites. Precise locations of individual groundwater wells are not shown because of confidentiality restrictions.

Groundwater was collected in autoclaved Nalgene bottles (Sigma-Aldrich, Saint-Louis, MO, United States) at the level of the surface separator for PDL samples (approximately 65 m below surface; reservoir temperature ~15 °C) and at the level of the surface orifice plate for SF samples (approximately 1,550 m below surface; reservoir temperature ~30 °C) during sampling campaigns conducted in February 2024 and February 2025 (Table 1). Both sampling campaigns were designed to assess the reproducibility of spatial community patterns across groundwater systems rather than to characterize temporal community dynamics.

**Table 1:** Groundwater samples included in this study.

Sample ID	Site	Well	Sampling date
PDL24_W1	Pointe-du-Lac	W1	2024-02-27
PDL24_W1-2	Pointe-du-Lac	W1	2024-02-27
PDL24_W2	Pointe-du-Lac	W2	2024-02-27
PDL24_W3	Pointe-du-Lac	W3	2024-02-27
PDL24_W4	Pointe-du-Lac	W4	2024-02-27
PDL24_W5	Pointe-du-Lac	W5	2024-02-27
PDL25_W1	Pointe-du-Lac	W1	2025-02-24
PDL25_W2	Pointe-du-Lac	W2	2025–01–29
PDL25_W2-II	Pointe-du-Lac	W2	2025-02-24
PDL25_W3	Pointe-du-Lac	W3	2025–01–29
PDL25_W3-II	Pointe-du-Lac	W3	2025-02-24
PDL25_W4	Pointe-du-Lac	W4	2025–01–29
PDL25_W4-II	Pointe-du-Lac	W4	2025-02-24
PDL25_W6	Pointe-du-Lac	W6	2025–01–29
PDL25_W7	Pointe-du-Lac	W7	2025–01–29
PDL25_W8	Pointe-du-Lac	W8	2025–01–29
PDL25_W8-II	Pointe-du-Lac	W8	2025-02-24
PDL25_W9	Pointe-du-Lac	W9	2025–01–29
PDL25_W9-II	Pointe-du-Lac	W9	2025-02-24
PDL25_W10-II	Pointe-du-Lac	W10	2025-02-24
PDL25_W11-II	Pointe-du-Lac	W11	2025-02-24
SF24-W1	Saint-Flavien	W1	2024-02-23
SF24-W2	Saint-Flavien	W2	2024-02-23
SF24-W3	Saint-Flavien	W3	2024-02-23
SF25_W1-hII	Saint-Flavien	W1	2025-02-18
SF25_W2-hII	Saint-Flavien	W2	2025-02-18
SF25_W3-hII	Saint-Flavien	W3	2025-02-18
SF25_W4	Saint-Flavien	W4	2025-02-18
SF25_W4-hII	Saint-Flavien	W4	2025-02-18
SF25_W5-hII	Saint-Flavien	W5	2025-02-18
SF25_W6	Saint-Flavien	W6	2025-02-18
SF25_W6-hII	Saint-Flavien	W6	2025-02-18

The table lists sample identifiers, study site, well designation, and sampling date for all groundwater samples collected from the Pointe-du-Lac (PDL) and Saint-Flavien (SF) groundwater systems and included in hydrochemical and microbial community analyses.

### 2.2 Environmental variable measurements

Each sample was drawn into a syringe that had been rinsed three times with the sample, then filtered through a 0.45 μm luer-lock syringe filter. Thirty mL was filtered for cation analysis and 15 mL for anion analysis. Samples were stored at −20 °C until analysis. Dissolved major and trace element concentrations were determined by inductively coupled plasma optical emission spectrometry (ICP-OES). Fifteen elements were quantified (Al, Be, Ca, Co, Cu, Fe, K, Mg, Mn, Na, Ni, Sb, Ti, V, and Zn), with three emission lines monitored per element. All solutions were prepared with ultrapure water (MilliQ). A 5% (v/v) nitric acid solution (HNO₃), used as both the calibration matrix and the instrument blank, was prepared by diluting 71.43 mL of 70% HNO₃ to 1 L with MilliQ water. Calibration relied on 2 multi-element stock standards in a 2% HNO₃ matrix. Six working standards were prepared by serial dilution of these stocks (and of intermediate standards) into 50 mL volumetric flasks, each brought to volume with the 5% HNO₃ solution and homogenized before transfer to falcon tubes. The full calibration series was prepared and measured in duplicate. Fifteen mL of each filtered sample was analyzed.

Seven anions were quantified (F^−^, Cl^−^, NO₂^−^, Br^−^, NO₃^−^, PO₄^3−^ and SO₄^2−^), each identified by retention time and quantified by peak area. All solutions were prepared with MilliQ water, which served as both the calibration diluent and the instrument blank. Separations were performed on an ion chromatograph fitted with an anion-exchange analytical column and a matching guard column, using a carbonate/bicarbonate eluent with suppressed conductivity detection. Calibration relied on 2 multi-anion standards in a MilliQ matrix. Six working standards were prepared by serial dilution of these standards (and of intermediate standards), each brought to volume with MilliQ water and homogenized before transfer to autosampler vials. The full calibration series was prepared and measured in duplicate. An aliquot of each filtered sample was transferred to a labeled autosampler vial for analysis.

### 2.3 DNA extraction, gene amplification, and sequencing

All groundwater samples were filtered through 0.2 μm filters (47 mm polyethersulfone, Sterlitech, Auburn, WA, United States), which were stored at −20 °C until extraction. DNA was extracted from the filters using the DNeasy PowerWater kit (Qiagen, Hilden, Germany). Bacterial and archaeal 16S rRNA gene fragments were amplified using the B341F (5′-CCTACGGGAGGCAGCAG-3′)/B785R (5′-GACTACHVGGGTATCTAATCC-3′) and A340F (5′-CCCTACGGGGYGCASCAG-3′)/A915R (5′-GTGCTCCCCCGCCAATTCCT-3′) primer pairs, respectively, with Phusion Hot Start II polymerase (Thermo Scientific, Waltham, MA, United States). PCR amplification was performed using annealing temperatures of 57 °C for bacterial reactions and 67 °C for archaeal reactions, with 35 amplification cycles for both domains.

Amplicon sequencing was performed on an Illumina MiSeq platform using MiSeq Reagent Kit v3 (600 cycles; Illumina, San Diego, CA, United States) at the CERMO-FC genomic platform (Centre d’excellence en recherche sur les maladies orphelines—Fondation Courtois, Université du Québec à Montréal, Canada). Quality control included one extraction blank processed alongside groundwater samples and PCR negative controls for both bacterial and archaeal amplifications, all of which were sequenced. The raw reads were deposited into the National Center for Biotechnology Information (NCBI) under the BioProject IDs PRJNA1481219 and PRJNA1481272.

### 2.4 Bioinformatic analyses

Raw bacterial and archaeal sequencing reads were processed separately using the DADA2 package in R v.4.2.0 to perform quality filtering, error correction, chimera removal, and generation of amplicon sequence variants (ASVs). Contaminant sequences were identified using reads obtained from extraction blanks and PCR negative controls and removed using the decontam package (method = “prevalence,” threshold = 0.5). Bacterial taxonomy was assigned using the SILVA v.138.1 reference database (Quast et al., 2013). Archaeal taxonomy was assigned using a custom reference database based on SILVA and supplemented with sequences from Liu et al. (2018) and Zhou et al. (2018).

For bacterial communities, a total of 977 ASVs were retained following sequence processing and contaminant removal. Samples containing fewer than 1,000 reads were excluded. To standardize sequencing effort across samples, bacterial communities were rarefied to the median sequencing depth, resulting in a final sequencing depth of 14,592 reads per sample. For archaeal communities, 19 samples yielded sequencing depths ranging from 3 to 1,957 reads. Because of the limited sequencing depth and uneven sample coverage, archaeal data were not rarefied and were interpreted conservatively. Archaeal analyses were therefore restricted to descriptive taxonomic characterization and exploratory comparison with bacterial community patterns.

### 2.5 Statistical analyses

All statistical analyses were performed in R. Community ecology analyses were performed using the vegan package. Unless otherwise specified, *p*-values were adjusted for multiple testing using the Benjamini–Hochberg (BH) procedure (p.adjust()) and considered significant at *p* < 0.05.

#### 2.5.1 Hydrochemical analyses

Hydrochemical variables were screened for completeness prior to analysis. Variables with substantial missing data were excluded from multivariate analyses. The remaining hydrochemical variables were centered and scaled (z-score standardization) prior to principal component analysis (PCA) to account for differences in units and concentration ranges among variables. Differences in hydrochemical composition between groundwater systems were visualized using PCA (prcomp()). Individual hydrochemical variables were compared between sites using Wilcoxon rank-sum tests (wilcox.test()). Within-site hydrochemical heterogeneity was evaluated using multivariate dispersion analyses (betadisper()). Relationships among hydrochemical variables were further explored using hierarchical clustering based on pairwise variable correlations (hclust()).

#### 2.5.2 Alpha-diversity analyses

Bacterial alpha diversity was quantified using observed ASV richness, Shannon diversity, and Pielou’s evenness, calculated using functions implemented in phyloseq and vegan. Differences between sites and sampling years were evaluated using Wilcoxon rank-sum tests, whereas differences among wells were assessed using Kruskal–Wallis tests (kruskal.test()).

#### 2.5.3 Beta-diversity analyses

Bacterial community composition was evaluated using Bray–Curtis dissimilarities calculated from relative abundance data (vegdist()). Ordinations were visualized using principal coordinates analysis (PCoA, cmdscale()). The effects of site, well identity, and sampling year on community composition were assessed using permutational multivariate analysis of variance (PERMANOVA, adonis2() with 9,999 permutations). Single-factor models were first evaluated independently, followed by marginal models to assess the contribution of individual variables after accounting for other factors. Homogeneity of multivariate dispersion was assessed using betadisper.

#### 2.5.4 Taxonomic composition and occupancy analyses

Genus-level relative abundances were calculated for all bacterial communities. Taxon prevalence was defined as the proportion of samples in which a genus was detected. Core microbiome analyses were conducted using occupancy-based classifications. Genera occurring in at least 75% of samples were classified as Core75 taxa, whereas genera occurring in at least 50% of samples were classified as Core50 taxa. Occupancy distributions were examined at both global and site-specific scales. Occupancy–abundance relationships were evaluated by plotting genus prevalence against mean relative abundance across samples. To quantify the relative contribution of persistent and non-persistent taxa to community structure, genera were grouped according to occupancy class, and their relative abundances were summed within each category.

#### 2.5.5 Hydrochemistry–microbiome relationships

Associations between bacterial community composition and hydrochemistry were evaluated using environmental fitting (envfit()) and distance-based redundancy analysis (db-RDA, capscale()), with significance assessed by permutation tests (9,999 permutations). Hydrochemical PCA scores were used as explanatory variables in db-RDA models. Global model significance and the significance of individual hydrochemical axes were assessed using permutation tests. The relative contributions of hydrochemistry and well identity to bacterial community structure were quantified using variance partitioning analyses (varpart()).

#### 2.5.6 Archaeal occurrence and bacterial–archaeal associations

Archaeal communities were analyzed descriptively because of low sequencing depth and variable archaeal detection among samples. Archaeal prevalence, taxonomic composition, and methanogen-associated signatures were summarized across wells and groundwater systems. Relationships between bacterial genera and methanogen-associated archaeal lineages were evaluated using Spearman rank correlations (cor.test()).

## 3 Results

### 3.1 Hydrochemical characterization of both groundwater habitats

#### 3.1.1 Site-level hydrochemical differentiation

Hydrochemical analyses were conducted after quality control filtering of environmental variables. Variables showing extensive missing values (PO₄^3−^, NO₂^−^, NO₃^−^, Cl^−^, and Br^−^) were excluded from multivariate analyses, while pH, conductivity, F^−^, SO₄^2−^ and dissolved elemental concentrations were retained (Supplementary Table 1).

PCA revealed clear hydrochemical differentiation between groundwater samples from Pointe-du-Lac (PDL) and Saint-Flavien (SF), with the first two principal components explaining 64.6% of total variation (Figure 2A; Supplementary Table 2). Site separation was primarily associated with contrasting elemental signatures rather than conductivity, pH, or sulfate. Along PC1, samples associated with higher Na, K, Mg, and Ca concentrations were opposed to samples associated with higher Al, Sb, Zn, Co, Cu, and Ni concentrations.

**Figure 2:**
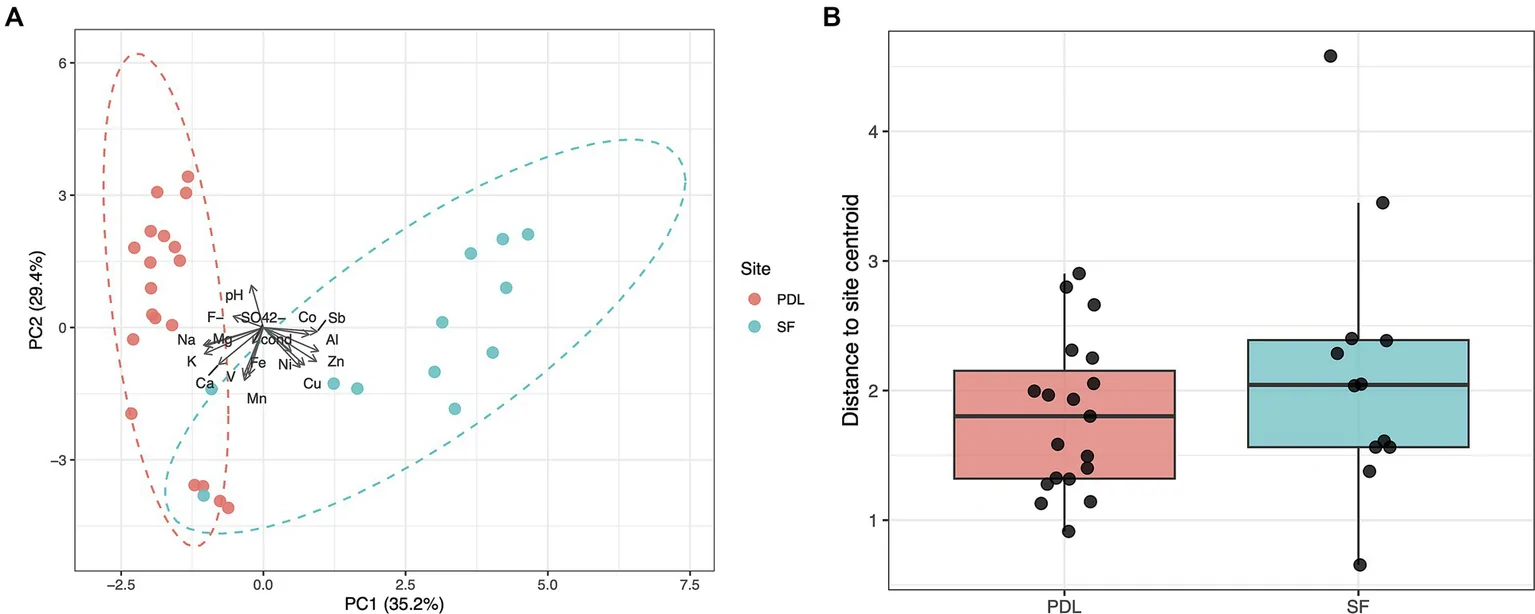
**(A)** Principal component analysis (PCA) of groundwater hydrochemistry showing the distribution of Pointe-du-Lac (PDL) and Saint-Flavien (SF) samples. Vectors indicate the contribution of hydrochemical variables to ordination structure. **(B)** Distance-to-centroid analysis showing within-site hydrochemical variability. Distances represent the multivariate distance of individual samples to the centroid of their respective groundwater system.

Univariate comparisons supported these multivariate patterns. After multiple-testing correction, PDL samples contained significantly higher concentrations of Na, K and Mg, whereas SF samples exhibited significantly higher concentrations of Al, Sb, Cu, Zn, Ni, and Co (Supplementary Table 3). Conductivity, pH, sulfate and fluoride did not differ significantly between sites.

#### 3.1.2 Within-site hydrochemical characteristics

Both sites displayed substantial within-site hydrochemical variability (Figure 2B). Median distance to the site centroid was slightly higher in SF than in PDL (2.04 and 1.80, respectively), but this difference was not significant (betadisper, *p* = 0.22).

#### 3.1.3 Environmental gradients and geochemical organization

Site-specific clustering indicated that hydrochemical organization differed between both groundwater systems. In PDL, major ions, pH and conductivity formed a broad integrated group, whereas trace elements constituted a secondary cluster and sulfate and fluoride remained comparatively isolated (Supplementary Figure 1A). In contrast, SF displayed a more compartmentalized structure, with dissolved ions, trace elements, conductivity-associated variables and anions organizing into more distinct groups (Supplementary Figure 1B).

### 3.2 Bacterial community structure in both groundwater samples

#### 3.2.1 Alpha diversity

After rarefaction, alpha diversity differed between groundwater systems for richness and evenness, but not for Shannon diversity (Figure 3A). Observed ASV richness was significantly higher in PDL than in SF (Wilcoxon test, BH-adjusted *p* = 0.0009). Evenness showed the opposite pattern and was significantly higher in SF than in PDL (BH-adjusted *p* = 0.0025). In contrast, Shannon diversity did not differ significantly between sites (BH-adjusted *p* = 0.4), indicating that differences in richness were not accompanied by detectable differences in abundance-weighted diversity.

**Figure 3:**
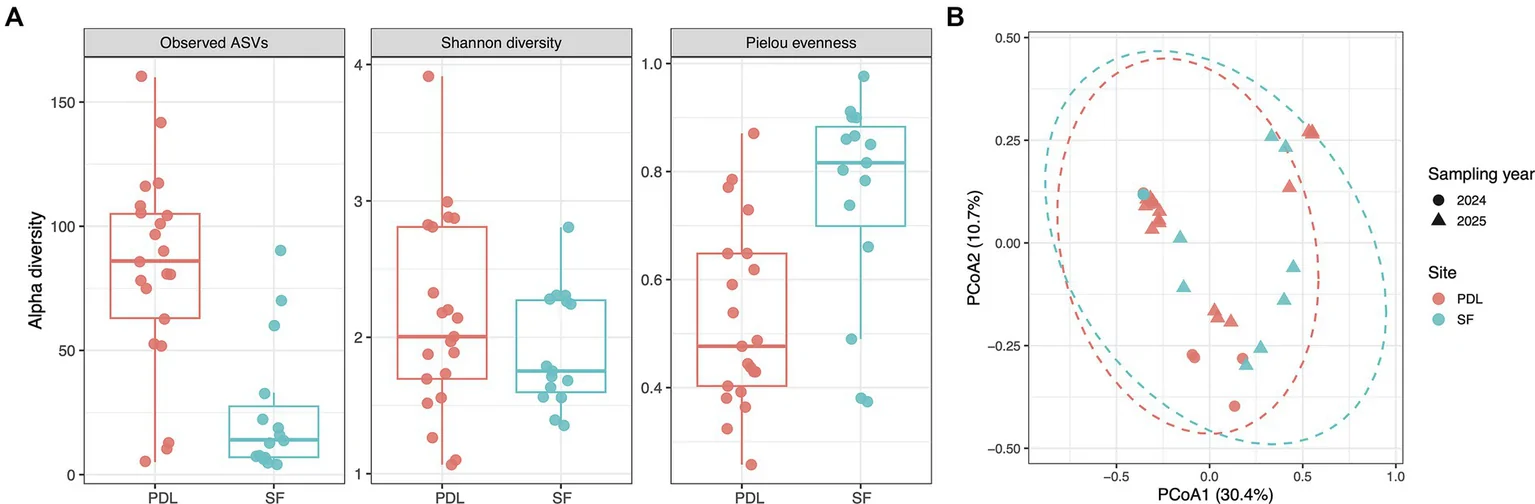
**(A)** Alpha-diversity metrics of bacterial communities in groundwater samples from Pointe-du-Lac (PDL) and Saint-Flavien (SF), including observed ASV richness, Shannon diversity, and Pielou evenness. Boxplots show median values and interquartile ranges, with individual points representing samples. **(B)** Principal coordinates analysis (PCoA) of bacterial community composition based on Bray–Curtis dissimilarities. Samples are colored by site and shaped according to sampling year.

No significant alpha-diversity differences were detected among wells after correction for multiple testing (Kruskal-Wallis test, observed ASVs: BH-adjusted *p* = 0.057; Shannon: BH-adjusted *p* = 0.085; evenness: BH-adjusted *p* = 0.11). Likewise, no significant differences in alpha diversity were detected between sampling years (Wilcoxon test, observed ASVs: BH-adjusted *p* = 0.37; Shannon: BH-adjusted *p* = 0.17; evenness: BH-adjusted *p* = 0.37).

#### 3.2.2 Beta diversity

Bacterial community composition showed substantial variation among groundwater samples, with PCoA capturing 41.1% of variation on the first two axes (Figure 3B).

In single-factor PERMANOVA models, site identity alone did not significantly explain bacterial community composition (Supplementary Table 4). In contrast, well identity explained a large fraction of community variation (*R*^2^ = 65.4%). Sampling year also explained a smaller but significant fraction of variation when tested alone (*R*^2^ = 6.7%).

In the marginal model, well identity remained significant (*p* = 0.001), while site did not contribute independently once well identity was accounted for Supplementary Table 5.

Dispersion analyses indicated that these compositional differences were not driven by unequal variance among groups. Dispersion did not differ significantly by site (betadisper, *p* = 0.54) or by well (*p* = 0.35).

Within-site analyses showed contrasting patterns. In PDL, well identity explained most bacterial variation (*R*^2^ = 75.7%), with a smaller significant year effect (*R*^2^ = 5.2%) (Supplementary Table 6). In SF, well identity was not significant, whereas sampling year explained a significant fraction of variation (*R*^2^ = 24.8%), although this result should be interpreted cautiously given the smaller number of wells and samples available from SF.

#### 3.2.3 Taxonomic overview

Bacterial communities were dominated by a limited number of genera, but dominant taxonomic profiles varied strongly among wells. Across wells-only samples, *Acetobacterium* was the dominant genus overall, with a mean relative abundance of 32.7%, and prevalence of 84.4% (Figure 4). *Polaromonas* was the second most abundant genus (mean relative abundance = 11.9%; prevalence = 71.9%), followed by *Allorhizobium*–*Neorhizobium*–*Pararhizobium*–*Rhizobium* (ANPR) (mean relative abundance = 6.6%; prevalence = 81.3%) and *Proteiniphilum* (mean relative abundance = 3.7%; prevalence = 65.6%).

**Figure 4:**
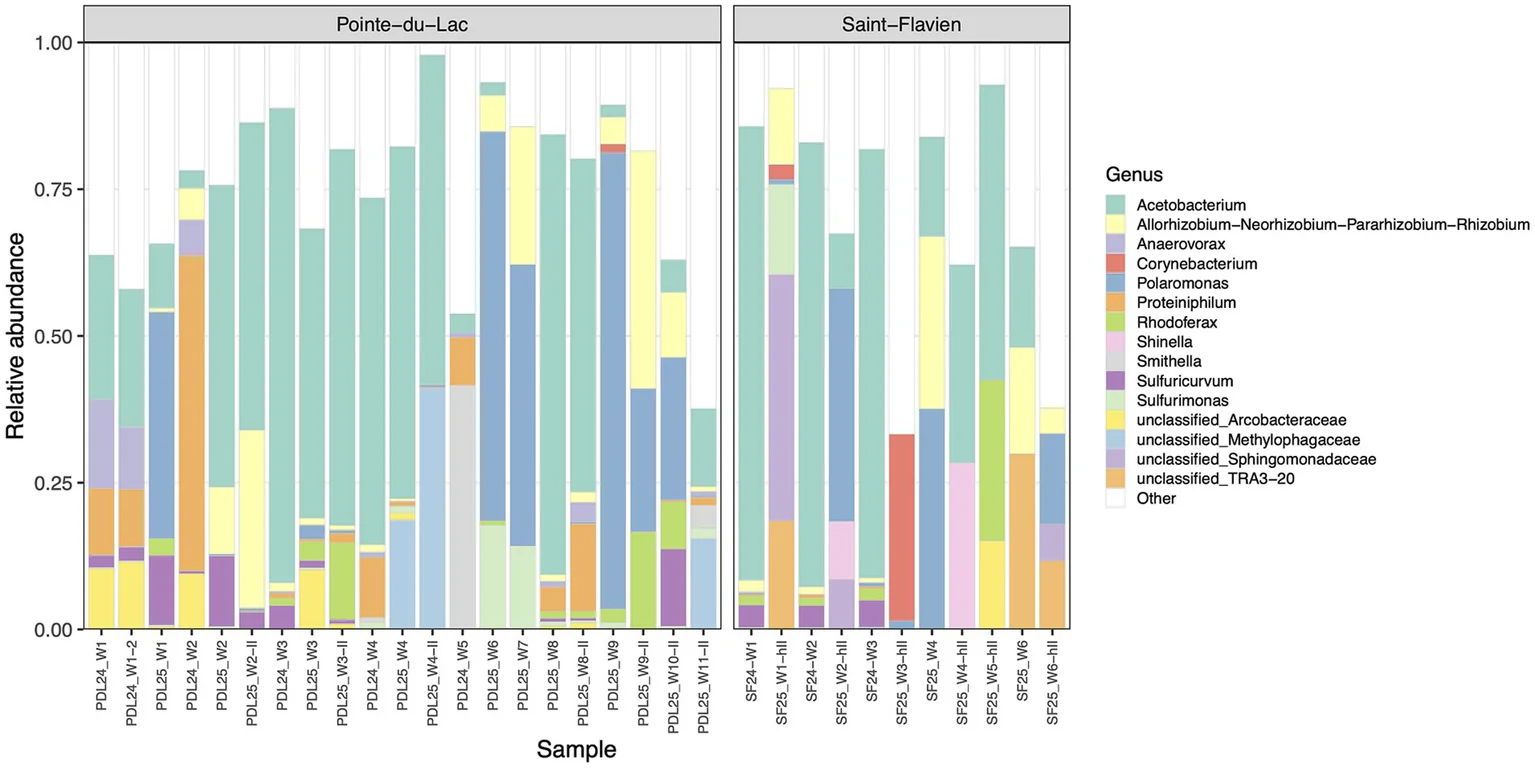
Relative abundance of the 15 most abundant bacterial genera across groundwater samples from Pointe-du-Lac (PDL) and Saint-Flavien (SF). Samples are grouped by groundwater system and displayed individually. Genera are shown as proportions of total community abundance, with all remaining taxa grouped as other.

Analysis of *Acetobacterium* ASVs showed that dominance was primarily associated with one lineage (Supplementary Figure 2). ASV1 accounted for most *Acetobacterium* reads, with 133,464 reads, a mean relative abundance of 28.6%, and a prevalence of 81.3% across groundwater samples. A secondary ASV was also recurrent but less dominant (ASV4), with 16,117 reads, a mean relative abundance of 3.5%, and a prevalence of 65.6%. Comparison of the two dominant *Acetobacterium* ASVs against the NCBI nucleotide database confirmed their affiliation with the genus *Acetobacterium*. Both ASV1 and ASV4 exhibited 100% sequence identity across the amplified 16S rRNA gene region to multiple described Acetobacterium species, including *A. malicum*, *A. wieringae*, and *A. carbinolicum* (Supplementary Table 7). Because several described species shared identical sequences across the amplified region, the available marker did not provide sufficient resolution to distinguish species-level lineages or ecotypes.

#### 3.2.4 Occupancy patterns and core microbiome structure

Occupancy distributions were strongly right-skewed, with most bacterial genera occurring in only a small fraction of groundwater samples (Supplementary Figure 3). Only a limited number of genera reached high prevalence across the dataset. Globally, 12 genera occurred in at least 50% of samples (Core50), whereas only two genera, *Acetobacterium* and ANPR, occurred in at least 75% of samples (Core75).

Core structure differed markedly between groundwater systems. PDL contained 29 Core50 genera, including nine Core75 genera: *Acetobacterium*, ANPR, *Proteiniphilum*, *Sulfurimonas*, *Anaerovorax*, *Fusibacter*, *Polaromonas*, *Desulfovibrio* and unclassified Actinobacteriota OPB41. In contrast, SF contained only three Core50 genera (*Acetobacterium*, *Polaromonas* and ANPR) and no Core75 genera.

#### 3.2.5 Non-core bacterial fraction

Occupancy–abundance analyses showed that only a small subset of genera combined high prevalence and high relative abundance across groundwater samples. Most genera occupied the low-prevalence and low-abundance portion of the distribution, indicating that the groundwater microbiome contained a large non-core component composed of infrequently detected taxa (Figure 5). The most abundant non-core genera included *Rhodoferax*, unclassified *Methylophagaceae*, *Sulfuricurvum*, unclassified *Arcobacteraceae*, *Smithella* and *Nocardioides*, which reached moderate relative abundances but occurred in fewer than 50% of samples.

**Figure 5:**
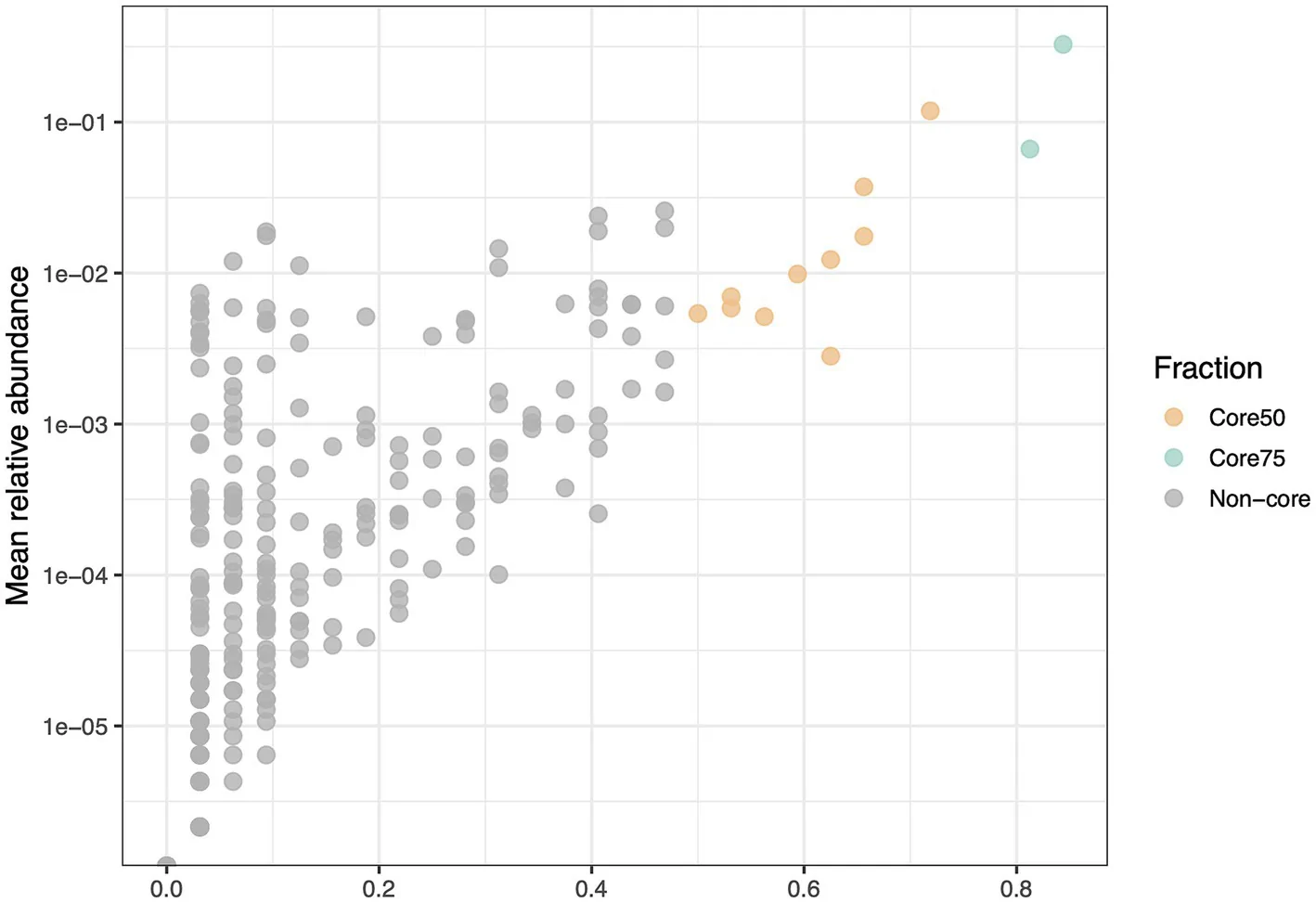
Occupancy–abundance relationship of bacterial genera across groundwater samples. Genus prevalence is plotted against mean relative abundance. Genera were classified as Core75 (detected in ≥75% of samples), Core50 (detected in 50–75% of samples), or non-core (<50% prevalence). The y-axis is shown on a logarithmic scale.

The relative contribution of non-core taxa differed between groundwater systems. In PDL, genera occurring in fewer than 50% of samples accounted for 32.4% of total community abundance. In contrast, non-core taxa accounted for 50% of total community abundance in SF. Consequently, persistent core taxa contributed proportionally more to community structure in PDL than in SF (Supplementary Figure 4). These results suggest that community organization differed between groundwater systems. PDL was structured around a relatively large and abundant recurrent core, whereas SF communities contained a greater contribution from low-occupancy taxa and a reduced persistent fraction.

### 3.3 Archaeal reads in both groundwater samples

Archaeal reads were detected in a subset of groundwater samples and showed marked differences between groundwater systems (Supplementary Figure 5). PDL contained substantially more archaeal-positive samples than SF, as well as higher archaeal read counts and greater archaeal diversity. Across archaeal-positive samples, PDL yielded 2,683 archaeal reads and a mean of 9.5 archaeal ASVs per positive sample, whereas SF yielded only 71 archaeal reads in total and a mean of one archaeal ASV per positive sample.

Taxonomic composition revealed a mixture of unresolved archaeal taxa and methanogen-associated lineages. The most abundant assignments included unclassified Archaea, unclassified *Thermoplasmata* and unclassified *Halobacterota*, indicating that much of the archaeal diversity could not be resolved beyond higher taxonomic ranks. Several methanogen-associated groups were repeatedly detected, including *Methanolobus*, *Methanocalculus*, members of the *Methanobacteriaceae* and *Methanocorpusculum* (Supplementary Figure 6). Rarity-profile analyses further indicated that these taxa recurred across multiple archaeal-positive samples rather than occurring as isolated detections.

Methanogen-associated signatures were concentrated in PDL and were absent from SF. Fourteen of fifteen archaeal-positive PDL samples contained at least one methanogen-associated lineage, with repeated detections observed. Several wells contained multiple methanogen-associated taxa simultaneously.

### 3.4 Hydrochemical drivers of bacterial community structure

To maximize overlap between hydrochemical and microbiome datasets, hydrochemical analyses were restricted to variables with sufficient data completeness across groundwater samples. The final dataset included pH, conductivity, SO₄^2−^, Al, Ca, Co, Cu, Fe, K, Mg, Mn, Na, Ni, Sb, V, and Zn, whereas variables with substantial missing values were excluded from multivariate analyses.

#### 3.4.1 Hydrochemical gradients associated with community turnover

Environmental fitting analyses identified several hydrochemical variables significantly associated with bacterial community composition (Supplementary Figure 7). pH showed the strongest association with community turnover (*r*^2^ = 0.51, *p* = 0.001), followed by Mn (*r*^2^ = 0.51, *p* = 0.002), V (*r*^2^ = 0.53, *p* = 0.003), Fe (*r*^2^ = 0.47, *p* = 0.005), K (*r*^2^ = 0.47, *p* = 0.005), and Ca (*r*^2^ = 0.31, *p* = 0.038). Together, these variables were significantly associated with bacterial compositional variation across groundwater wells.

#### 3.4.2 Hydrochemistry gradients associated with community structure

PCA of groundwater chemistry revealed two dominant hydrochemical gradients (Figure 6). ChemPC1 explained 40.2% of hydrochemical variation and ChemPC2 explained 33.0%, together accounting for 73.2% of total hydrochemical variance. ChemPC1 contrasted waters enriched in Na, Mg, Ca, K, Fe, Mn, and V against waters characterized by higher Al, Sb, Ni, Co, and Zn concentrations.

**Figure 6:**
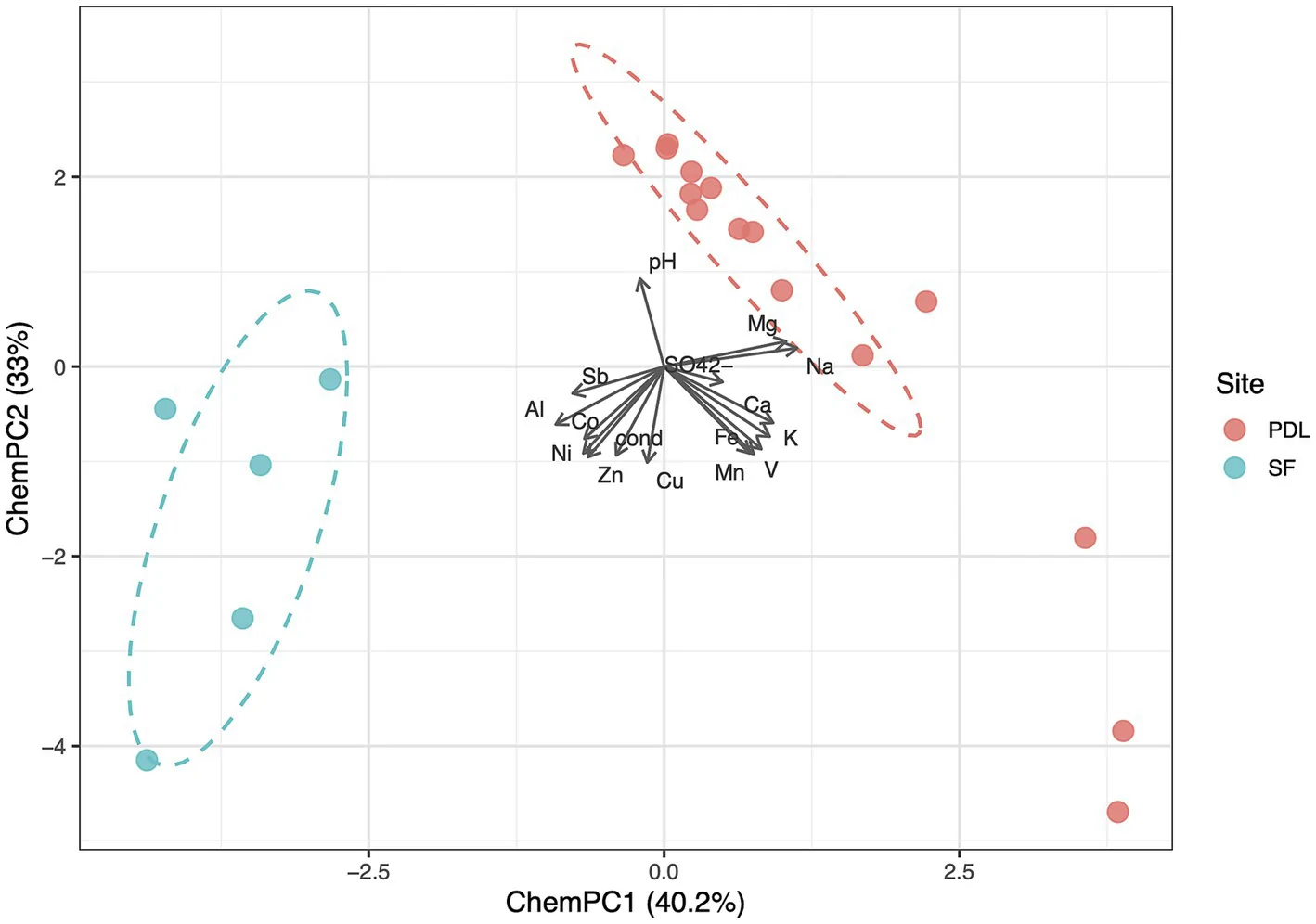
Principal component analysis (PCA) of groundwater hydrochemistry used to generate the hydrochemical gradients (ChemPC1 and ChemPC2) included in subsequent hydrochemistry–microbiome analyses. The PCA was based on the subset of hydrochemical variables retained for microbial community analyses. Vectors indicate the contribution of individual hydrochemical variables to ordination structure.

Db-RDA demonstrated a significant relationship between hydrochemistry and bacterial community composition (Figure 7; Supplementary Table 8). Both hydrochemical axes contributed significantly to bacterial turnover, indicating that bacterial community differentiation was associated with multiple hydrochemical dimensions rather than a single environmental gradient.

**Figure 7:**
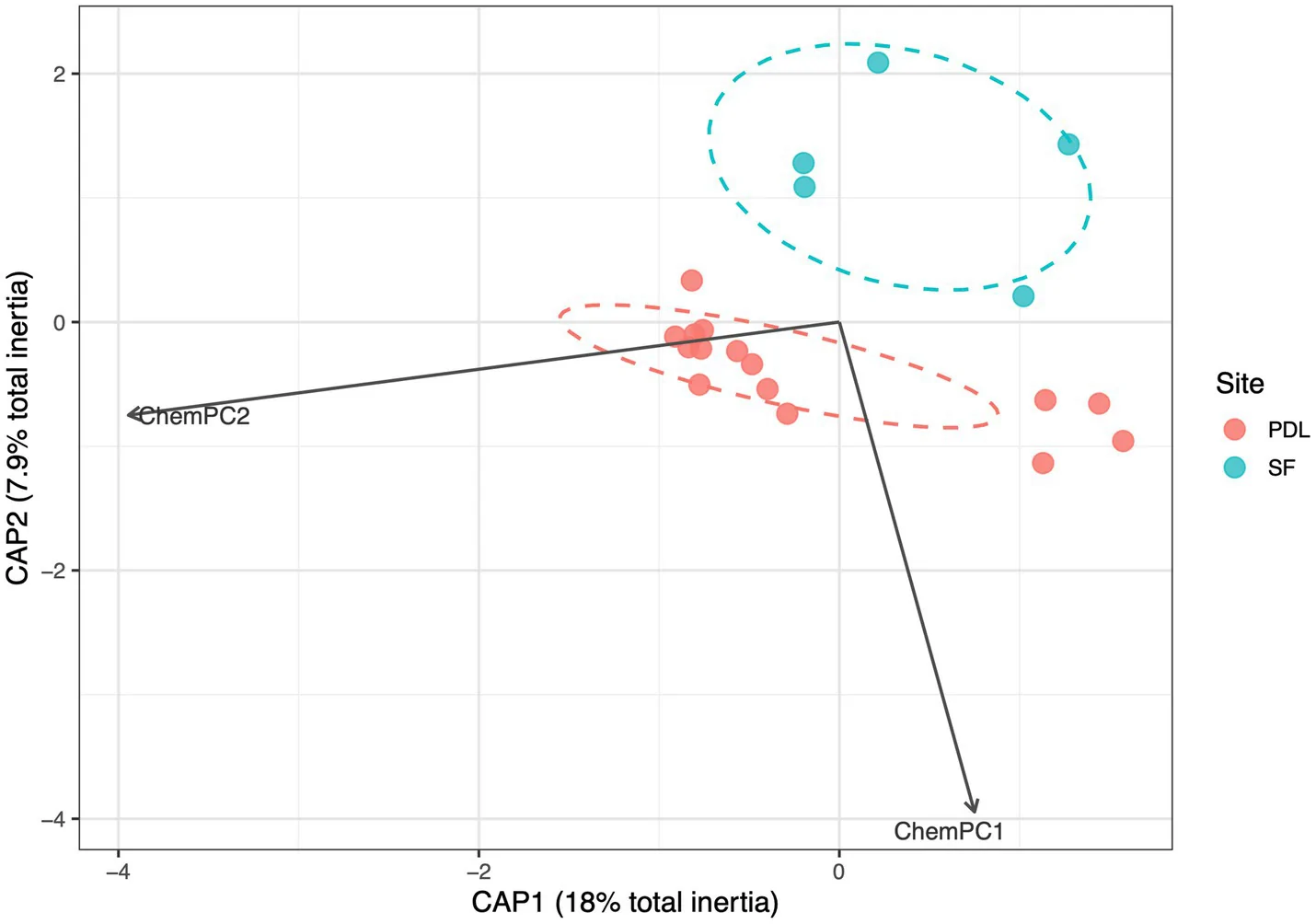
Distance-based redundancy analysis (db-RDA) showing the relationship between groundwater hydrochemistry and bacterial community composition. Samples are colored according to groundwater system and constrained by the two hydrochemical PCA axes used in the model.

Variance partitioning indicated that well identity accounted for the largest independent fraction of bacterial community variation (43.8%), whereas hydrochemistry explained a smaller independent fraction (12.3%). Only 5.2% of variation was jointly explained by well identity and hydrochemistry. Partitioning hydrochemistry into individual chemistry axes showed that ChemPC2 explained a larger fraction of variation (13.2%) than ChemPC1 (2.4%). These results indicate that although hydrochemistry contributed to bacterial community structure, well identity remained the dominant source of ecological differentiation among groundwater communities.

#### 3.4.3 Hydrochemical associations with dominant taxa

Taxon–environment analyses identified a limited number of bacterial genera strongly associated with hydrochemical gradients. *Proteiniphilum* showed the strongest association with hydrochemistry (Spearman, ρ = 0.827) (Supplementary Table 9). Significant correlations were also detected for *Acetobacterium* (ρ = 0.669), *Desulfovibrio* (ρ = 0.671), *Polaromonas* (ρ = 0.645), and an unclassified member of the *Methylophagaceae* (ρ = 0.705).

Within-site analyses showed that several associations remained significant within PDL, including correlations involving *Acetobacterium*, *Proteiniphilum* and *Polaromonas*. In contrast, no significant within-site correlations were detected in SF after correction for multiple testing. These results indicate that hydrochemistry influenced bacterial community composition, but that these effects were concentrated within a relatively small subset of dominant taxa rather than being distributed uniformly across the entire community.

### 3.5 Relationships between bacterial and archaeal community structure

Correlation analyses identified numerous positive associations between bacterial taxa and methanogen-associated archaeal signatures (Supplementary Table 10). Many of the strongest correlations involved relatively low-abundance anaerobe-associated taxa, including *Anaerovorax*, Candidatus *Deferrimonas*, *Desulfomicrobium* and members of the *Synergistaceae*. Among the dominant bacterial genera, *Proteiniphilum* showed a strong positive association with unclassified *Methanobacteriaceae* (Spearman, ρ = 0.76), whereas *Acetobacterium* did not retain significance after Benjamini–Hochberg correction. These results indicate that methanogen-associated archaeal signatures were associated primarily with a limited subset of anaerobe-associated bacterial taxa rather than with broad community-wide shifts.

## 4 Discussion

### 4.1 Deep groundwater microbial communities remained strongly well-specific despite contrasting hydrochemical environments

One of the most striking patterns observed in this study was the predominance of well identity over broader groundwater-system effects. Although Pointe-du-Lac (PDL) and Saint-Flavien (SF) differed substantially in hydrochemical composition, bacterial communities were structured primarily at the scale of individual wells rather than by groundwater system. Well identity consistently explained the largest fraction of community variation, whereas site identity did not contribute independently once well-level variation was considered. Although both sites represent subsurface groundwater habitats, they differ markedly in depth and hydrogeological context, with PDL representing a comparatively shallow groundwater system and SF corresponding to deep formation waters.

The contrasting hydrogeological characteristics of both systems may help explain this strong well-specific structuring. PDL consists of highly permeable unconsolidated sands and gravels that remain hydraulically connected to an underlying saline aquifer, whereas SF occurs within a deep fractured dolomite reservoir where permeability is controlled primarily by fracture networks and hydraulic support appears limited. Such differences in physical connectivity likely influence microbial dispersal, groundwater residence times, and resource exchange, creating localized habitats that are only partially reflected by measured hydrochemical variables.

Rather than behaving as homogeneous groundwater bodies, both systems appear to consist of multiple localized ecological habitats. These results support the view that microbial communities in groundwater are often organized primarily by local hydrogeological context, with groundwater flow paths, residence time, and hydrological isolation exerting strong influences on community assembly (Ben Maamar et al., 2015; Fillinger et al., 2019). Restricted connectivity among wells may further limit microbial dispersal, promoting the development of distinct well-specific communities even within the same groundwater system (Fillinger et al., 2019). Similar patterns have recently been observed in long-term groundwater studies, where hydrologically isolated aquifers exhibited distinct microbial assemblages compared with more connected groundwater systems (Wang et al., 2025). Although Wang et al. (2025) examined shallow carbonate aquifers, the consistency of these patterns across systems differing markedly in depth and geological setting suggests that hydrogeological connectivity may represent a general driver of groundwater microbial community organization.

### 4.2 Hydrochemistry acted as an environmental filter within localized groundwater habitats

Despite the dominance of well-specific effects, hydrochemistry remained an important source of ecological differentiation. Several hydrochemical variables, including pH, Fe, Mn, V, K, and Ca, were significantly associated with bacterial turnover, and hydrochemical gradients explained a significant fraction of community variation in db-RDA analyses. Hydrochemical gradients are widely recognized as important drivers of groundwater microbial communities because they influence energy availability, redox conditions, and nutrient distributions within subsurface habitats (Griebler and Lueders, 2009; Hug et al., 2015).

The two groundwater systems displayed contrasting geochemical signatures. PDL was characterized by elevated concentrations of major ions including Na, K, Mg, and Ca, whereas SF was enriched in trace elements such as Al, Sb, Co, Ni, Cu, and Zn. However, hydrochemistry explained only a modest fraction of bacterial variation relative to well identity, indicating that environmental filtering occurred within already differentiated groundwater habitats rather than acting as the primary determinant of community composition. Similar patterns have been reported in other groundwater systems, where hydrochemical conditions influence community composition but explain less variation than hydrogeological isolation and local habitat structure (Ben Maamar et al., 2015; Fillinger et al., 2019). Although a broad suite of hydrochemical variables was characterized, additional hydrological, geological, and hydrogeological factors not measured in this study may contribute to the substantial fraction of community variation that remained unexplained.

The differing hydrochemical organization of the two systems may nevertheless provide insight into broader ecological differences. PDL exhibited a relatively integrated geochemical structure in which major ions, conductivity and pH covaried, whereas SF displayed a more compartmentalized organization involving distinct trace-element and ion clusters. Such differences may reflect contrasting groundwater connectivity, fluid circulation or geochemical evolution. Although these processes cannot be directly resolved from the present dataset, they are consistent with the contrasting microbial organization observed between systems. Consistent with this interpretation, only a limited number of dominant bacterial taxa exhibited strong associations with hydrochemical gradients. This suggests that environmental filtering acted on specific lineages rather than uniformly across the entire community, reinforcing the view that hydrochemistry contributed to community differentiation without fully determining microbial community structure. Overall, hydrochemistry appeared to function as a secondary ecological filter acting within hydrogeologically structured habitats rather than as the dominant driver of microbial community assembly.

Although a broad suite of hydrochemical variables was characterized, 38.7% of bacterial community variation remained unexplained by well identity and measured hydrochemistry. This residual variation may reflect unmeasured hydrological and geological properties, including groundwater residence time, local flow paths, fracture connectivity, and microscale physicochemical heterogeneity, as well as ecological processes not represented by the measured environmental variables. Viewed within the broader framework of microbial community assembly, our results are consistent with contributions from both deterministic and stochastic processes. The significant associations between hydrochemical gradients and bacterial composition support deterministic environmental filtering, whereas strong well-specific structuring may reflect dispersal limitation and historical contingency associated with hydrogeological compartmentalization. Stochastic demographic processes and fine-scale microhabitat heterogeneity may additionally contribute to variation among communities. However, because the present analyses were not designed to explicitly partition deterministic and stochastic assembly processes, the relative contributions of these mechanisms cannot be quantified from the current dataset.

### 4.3 *Acetobacterium* dominance suggests recurring strategies for persistence in energy-limited groundwater habitats

A central biological feature of both groundwater systems was the widespread dominance of *Acetobacterium*. This genus represented the most abundant bacterial lineage across the dataset, occurred in most groundwater samples, and was one of only two genera detected in at least 75% of all samples. Dominance was largely associated with a single recurrent ASV that was distributed across multiple wells and both groundwater systems. Given the strong well-specific structuring observed throughout the dataset, the widespread occurrence of this lineage across geographically separated and hydrochemically distinct habitats suggests an exceptional capacity to persist under contrasting groundwater conditions and may reflect adaptation to ecological constraints common to energy-limited subsurface environments.

*Acetobacterium* species are commonly associated with acetogenic metabolism and have frequently been reported from anoxic and energy-limited habitats where reduced compounds and hydrogen can support carbon fixation through the Wood–Ljungdahl pathway (Schuchmann and Müller, 2016). Acetogenic microorganisms are increasingly recognized as important components of subsurface ecosystems because their metabolic flexibility enables growth under conditions of low energy availability and limited organic carbon inputs (Lever, 2012). Although metabolic activity cannot be inferred from amplicon data alone, the widespread distribution of this lineage is consistent with adaptation to conditions common in oligotrophic groundwater systems.

Notably, *Acetobacterium* was significantly associated with hydrochemical gradients but did not emerge as the strongest predictor of methanogen-associated archaeal occurrence. This pattern suggests that its success may be linked more closely to broad groundwater habitat characteristics than to specific archaeal assemblages. The recurrence of a dominant *Acetobacterium* lineage across both groundwater systems therefore points toward the existence of shared ecological constraints that favor particular metabolic strategies in deep groundwater habitats. Similar recurring acetogenic populations have been reported from geographically distinct subsurface environments, suggesting that a limited set of ecological strategies may repeatedly emerge under chronic energy limitation (Magnabosco et al., 2018; Momper et al., 2017).

### 4.4 Ecological characteristics of recurrent and localized groundwater taxa

Although *Acetobacterium* represented the most conspicuous bacterial lineage across both groundwater systems, the broader composition of recurrent taxa suggests that different ecological conditions may characterize PDL and SF. In PDL, the persistent community included several taxa commonly associated with anaerobic and reduced environments, including *Proteiniphilum*, *Anaerovorax*, *Fusibacter*, *Desulfovibrio*, *Desulfomicrobium*, *Sulfurimonas*, *Sulfuricurvum,* and *Dethiosulfatibacter*. Many of these genera are linked to fermentative metabolisms, sulfur cycling, or other anaerobic processes that have frequently been reported from subsurface and energy-limited habitats (Griebler and Lueders, 2009; Magnabosco et al., 2018). Although amplicon sequencing does not provide direct evidence of metabolic activity, the repeated occurrence of these taxa across multiple wells suggests that reduced conditions may favor bacterial taxa commonly associated with anaerobic metabolic strategies within the PDL groundwater ecosystem. The higher richness observed in PDL, together with its larger recurrent bacterial component, further suggests that this groundwater system may support a broader range of ecological niches than SF despite similar abundance-weighted diversity.

In contrast, SF was characterized by a much smaller recurrent bacterial component dominated by *Acetobacterium*, *Polaromonas,* and ANPR, while many other taxa occurred only sporadically or were restricted to individual wells. Unlike many of the recurrent taxa observed in PDL, *Polaromonas* is frequently reported from oligotrophic groundwater and aquatic environments and is often considered a metabolically versatile lineage capable of persisting across a wide range of environmental conditions (Mattes et al., 2008; Wilhelm et al., 2011). Its widespread occurrence in both groundwater systems, combined with its strong association with hydrochemical gradients, suggests that ecological persistence in these habitats is not restricted to strictly anaerobic specialists but may also involve broadly adapted groundwater-associated taxa. Several of the more localized taxa detected in SF, including *Rhodoferax*, *Nocardioides*, and members of the *Sphingomonadaceae*, are similarly associated with metabolically flexible environmental populations and did not form the coherent recurrent assemblage observed in PDL.

The contrasting taxonomic organization of the two groundwater systems therefore extended beyond taxonomic composition alone. PDL supported a recurrent assemblage enriched in anaerobic and sulfur-associated lineages, whereas SF appeared to be structured around a smaller set of persistent taxa and a larger proportion of localized populations. These observations suggest that the two systems may represent distinct modes of subsurface ecosystem organization, potentially reflecting differences in groundwater connectivity, habitat compartmentalization and long-term environmental stability.

### 4.5 Methanogen-associated archaeal signatures were restricted to a subset of groundwater habitats

Archaeal communities were detected primarily in PDL and were largely absent from SF, therefore preventing robust comparisons of archaeal community composition between sites. Although archaeal sequencing depth remained very limited, repeated occurrence of methanogen-associated lineages across multiple wells suggests that methanogenic archaea form a recurring component of certain groundwater habitats. Methanogenic archaea are widely reported from anoxic groundwater and deep subsurface environments, although their abundance and distribution are often highly heterogeneous at local spatial scales (Moser et al., 2005). Several methanogen-associated groups were repeatedly detected, including *Methanolobus*, *Methanocalculus*, *Methanobacteriaceae,* and *Methanocorpusculum*. The presence of multiple methanogenic lineages within the same wells suggests that methanogen-associated signatures were not restricted to isolated taxonomic detections but instead occurred within localized archaeal assemblages. The concentration of these signatures in a limited subset of wells further indicates that suitable niches for methanogenic populations were unevenly distributed throughout the groundwater system.

The methanogen-associated signatures detected in PDL also encompassed multiple methanogenic lineages with distinct metabolic strategies. *Methanolobus*, the most recurrent methanogen-associated genus, is typically associated with methylotrophic methanogenesis, whereas *Methanocalculus*, *Methanobacteriaceae* and *Methanocorpusculum* are generally linked to hydrogenotrophic pathways (Liu and Whitman, 2008; Borrel et al., 2013). Although amplicon sequencing cannot be used to infer active metabolism, the repeated detection of both methylotrophic and hydrogenotrophic methanogens suggests that multiple methanogenic niches may coexist within these groundwater habitats. This diversity of potential methanogenic strategies is consistent with the geochemical and ecological heterogeneity observed among wells and further supports the existence of localized anaerobic microenvironments within the PDL groundwater system.

Relationships between methanogen-associated archaeal signatures and bacterial taxa were similarly localized. Positive associations were observed with several anaerobe-associated bacterial groups, while *Proteiniphilum* emerged as the strongest association among dominant bacterial genera. These patterns are consistent with the occurrence of localized reduced microhabitats that support both methanogenic archaea and subsets of anaerobic bacteria. However, the correlation-based nature of these analyses, together with the low archaeal sequencing depth, precludes inference of direct ecological interactions.

### 4.6 Challenges and opportunities for studying deep subsurface microbial ecosystems

Studies of deep subsurface microbial ecosystems remain constrained by the inherent challenges of accessing groundwater habitats that are physically isolated, spatially heterogeneous, and frequently characterized by low microbial biomass (Magnabosco et al., 2018; Griebler and Lueders, 2009). These limitations are particularly evident in archaeal datasets, where low read recovery can restrict statistical analyses despite the ecological importance of methanogenic populations. As a result, many subsurface environments remain poorly characterized relative to surface ecosystems, and fundamental questions regarding microbial persistence, dispersal, and metabolic activity remain unresolved. Despite these challenges, the present study demonstrates that habitat-centered approaches can provide valuable insight into the ecological organization of subsurface microbial communities. By integrating hydrochemistry, bacterial and archaeal community composition, and occupancy-based analyses across contrasting groundwater systems, we identified consistent patterns of well-specific community assembly, environmental filtering, and microbial persistence. These findings suggest that understanding the deep biosphere requires consideration not only of geochemical conditions but also of the hydrogeological context in which microbial habitats occur. As access to subsurface environments increases and sequencing approaches continue to improve, combining community surveys with metagenomics, geochemical monitoring, and direct measurements of microbial activity will provide a more complete understanding of how microbial life persists in energy-limited groundwater ecosystems (Momper et al., 2017). Such efforts will be essential for resolving the ecological and biogeochemical processes that govern microbial communities across the deep subsurface.

An additional consideration is that both groundwater systems are associated with active underground natural gas storage facilities and therefore cannot be considered completely undisturbed subsurface environments. Periodic gas injection and withdrawal may alter reservoir pressure, fluid movement, and gas–water interfaces and could consequently influence local hydrochemical conditions, microbial dispersal, or the introduction of microorganisms from operational infrastructure. The extent to which these processes contributed to the community patterns observed here cannot be resolved from the present dataset. Nevertheless, the reproducible spatial patterns observed, particularly the strong well-specific structuring in PDL, suggest that these groundwater habitats retain localized ecological signatures despite their operational context. Our findings should therefore be interpreted as characterizing microbial community organization within subsurface groundwater systems used for natural gas storage, and extrapolation to undisturbed natural aquifers should be made cautiously.

## 5 Conclusion

Deep groundwater microbial communities were structured primarily at the scale of individual wells rather than by broad hydrochemical differences between groundwater systems. Although hydrochemistry contributed significantly to community turnover, well identity consistently explained a larger fraction of bacterial community variation, highlighting the importance of local habitat conditions and hydrogeological compartmentalization in shaping subsurface ecosystems. The two groundwater systems differed markedly in microbial persistence patterns and community organization. PDL supported a larger recurrent bacterial component, stronger taxon–environment relationships, and repeated methanogen-associated archaeal signatures, whereas SF contained a smaller persistent fraction and a greater contribution from localized taxa. The widespread dominance of a single *Acetobacterium* lineage across both systems further suggests that a limited number of ecological strategies may be particularly successful in oligotrophic groundwater habitats. These results indicate that deep groundwater ecosystems are shaped by the combined effects of hydrogeological isolation, environmental filtering, and the persistence of specialized microbial lineages adapted to energy-limited conditions.

## Data Availability

The raw reads generated in this study were deposited into the National Center for Biotechnology Information (NCBI) under the BioProject IDs PRJNA1481219 and PRJNA1481272.
