# Supplementary material for: Hydrogeological compartmentalization outweighs hydrochemistry in structuring microbial communities across contrasting subsurface habitats
Source: Front Microbiol. 2026 Sep 28;17:1919949. doi: 10.3389/fmicb.2026.1919949 (PMC13656802; doi:10.3389/fmicb.2026.1919949)
Supplement: Supplementary file 1 [file Data_sheet_1.zip › Supplementary Material/Figure S1A.pdf]

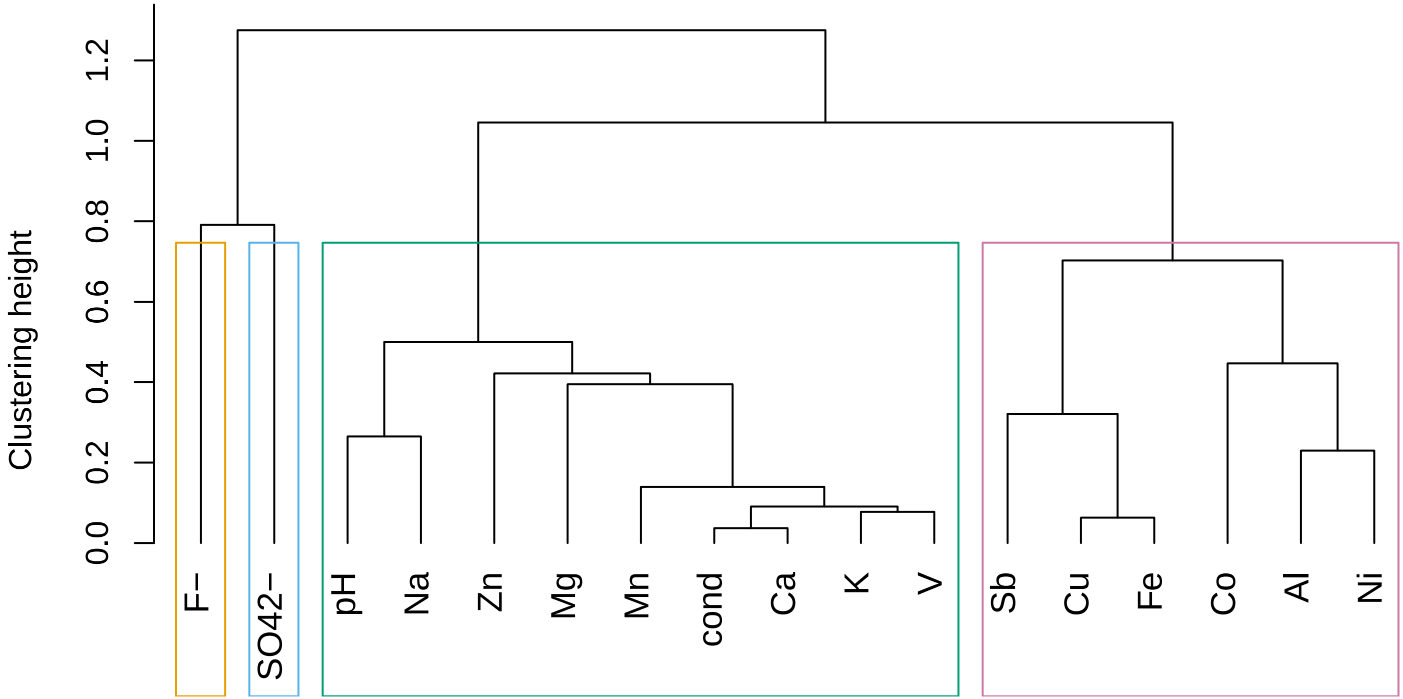
Clustering height
0.0
0.2
0.4
0.6
0.8
1.0
1.2
F−
SO42−
pH
Na
Zn
Mg
Mn
cond
Ca
K
V
Sb
Cu
Fe
Co
Al
Ni

SM Figure 1A
