# Supplementary material for: Hydrogeological compartmentalization outweighs hydrochemistry in structuring microbial communities across contrasting subsurface habitats
Source: Front Microbiol. 2026 Sep 28;17:1919949. doi: 10.3389/fmicb.2026.1919949 (PMC13656802; doi:10.3389/fmicb.2026.1919949)
Supplement: Supplementary file 1 [file Data_sheet_1.zip › Supplementary Material/Figure S1B.pdf]

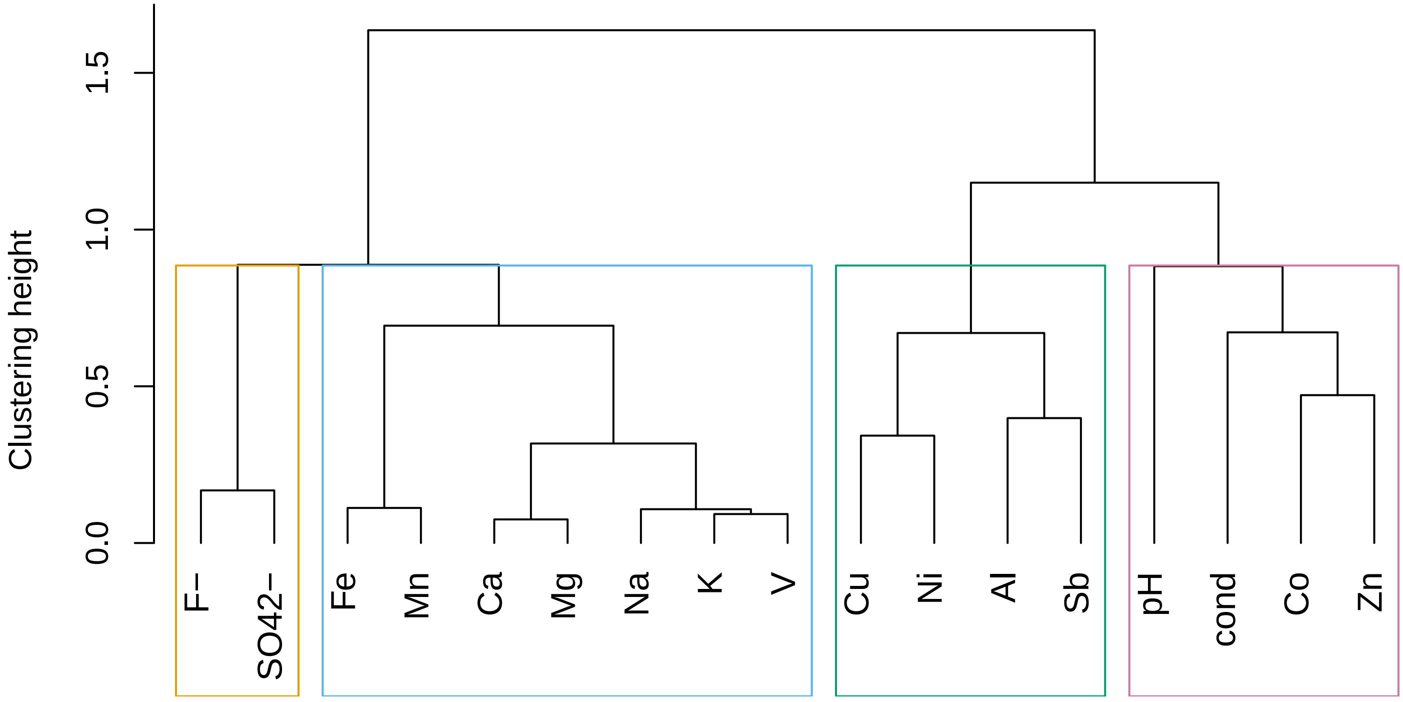

Clustering height
0.0
0.5
1.0
1.5
F−
SO42−
Fe
Mn
Ca
Mg
Na
K
V
Cu
Ni
Al
Sb
pH
cond
Co
Zn

SM Figure 1B
