# Supplementary material for: Hydrogeological compartmentalization outweighs hydrochemistry in structuring microbial communities across contrasting subsurface habitats
Source: Front Microbiol. 2026 Sep 28;17:1919949. doi: 10.3389/fmicb.2026.1919949 (PMC13656802; doi:10.3389/fmicb.2026.1919949)
Supplement: Supplementary file 1 [file Data_sheet_1.zip › Supplementary Material/Figure S2.pdf]

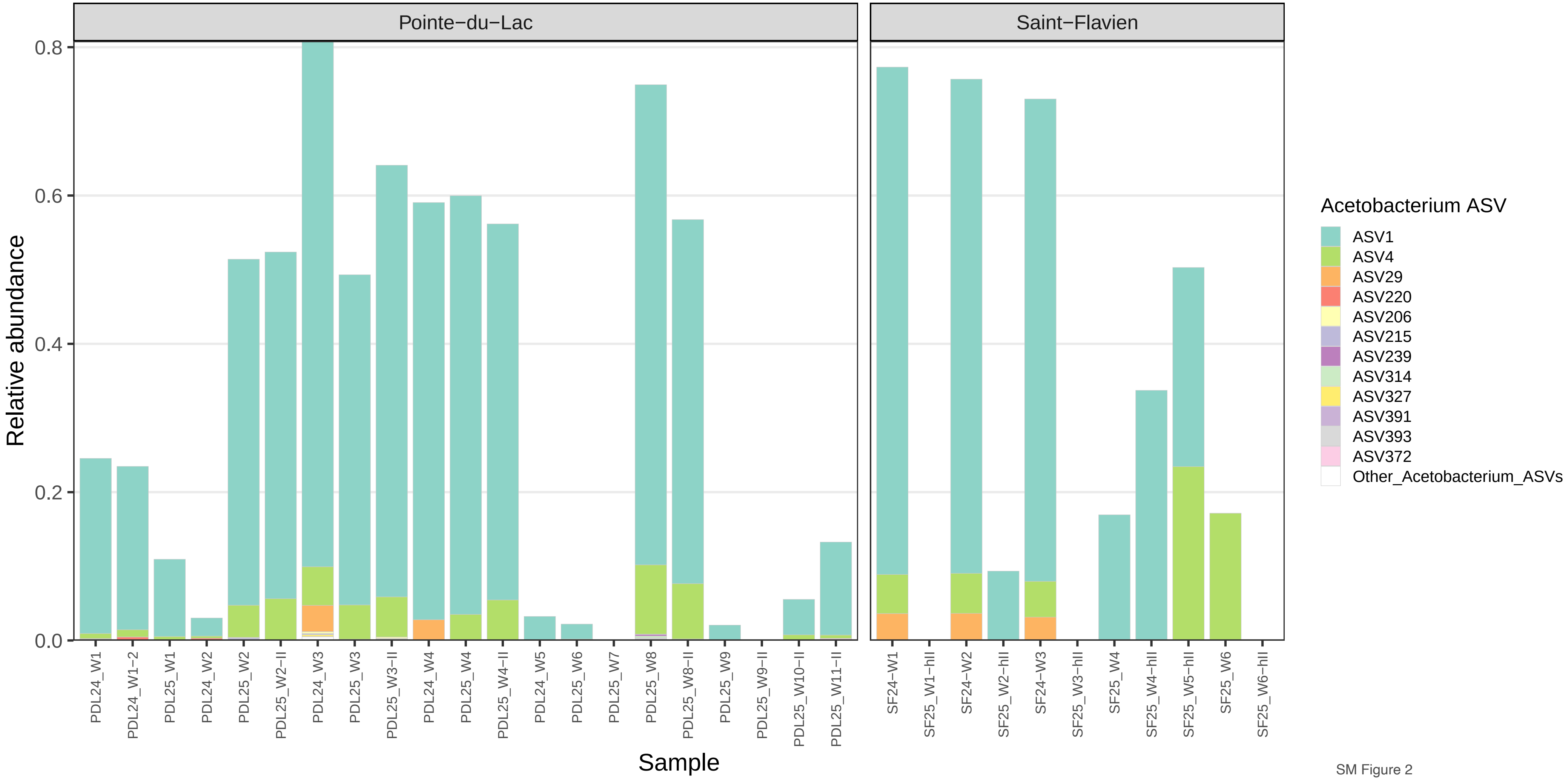

Pointe−du−Lac
Saint−Flavien
Relative abundance
0.0
0.2
0.4
0.6
0.8
PDL24_W1
PDL24_W1−2
PDL25_W1
PDL24_W2
PDL25_W2
PDL25_W2−II
PDL24_W3
PDL25_W3
PDL25_W3−II
PDL24_W4
PDL25_W4
PDL25_W4−II
PDL24_W5
PDL25_W6
PDL25_W7
PDL25_W8
PDL25_W8−II
PDL25_W9
PDL25_W9−II
PDL25_W10−II
PDL25_W11−II
SF24−W1
SF25_W1−hII
SF24−W2
SF25_W2−hII
SF24−W3
SF25_W3−hII
SF25_W4
SF25_W4−hII
SF25_W5−hII
SF25_W6
SF25_W6−hII
Sample
Acetobacterium ASV
ASV1
ASV4
ASV29
ASV220
ASV206
ASV215
ASV239
ASV314
ASV327
ASV391
ASV393
ASV372
Other_Acetobacterium_ASVs

SM Figure 2
