# Supplementary material for: Hydrogeological compartmentalization outweighs hydrochemistry in structuring microbial communities across contrasting subsurface habitats
Source: Front Microbiol. 2026 Sep 28;17:1919949. doi: 10.3389/fmicb.2026.1919949 (PMC13656802; doi:10.3389/fmicb.2026.1919949)
Supplement: Supplementary file 1 [file Data_sheet_1.zip › Supplementary Material/Figure S3.pdf]

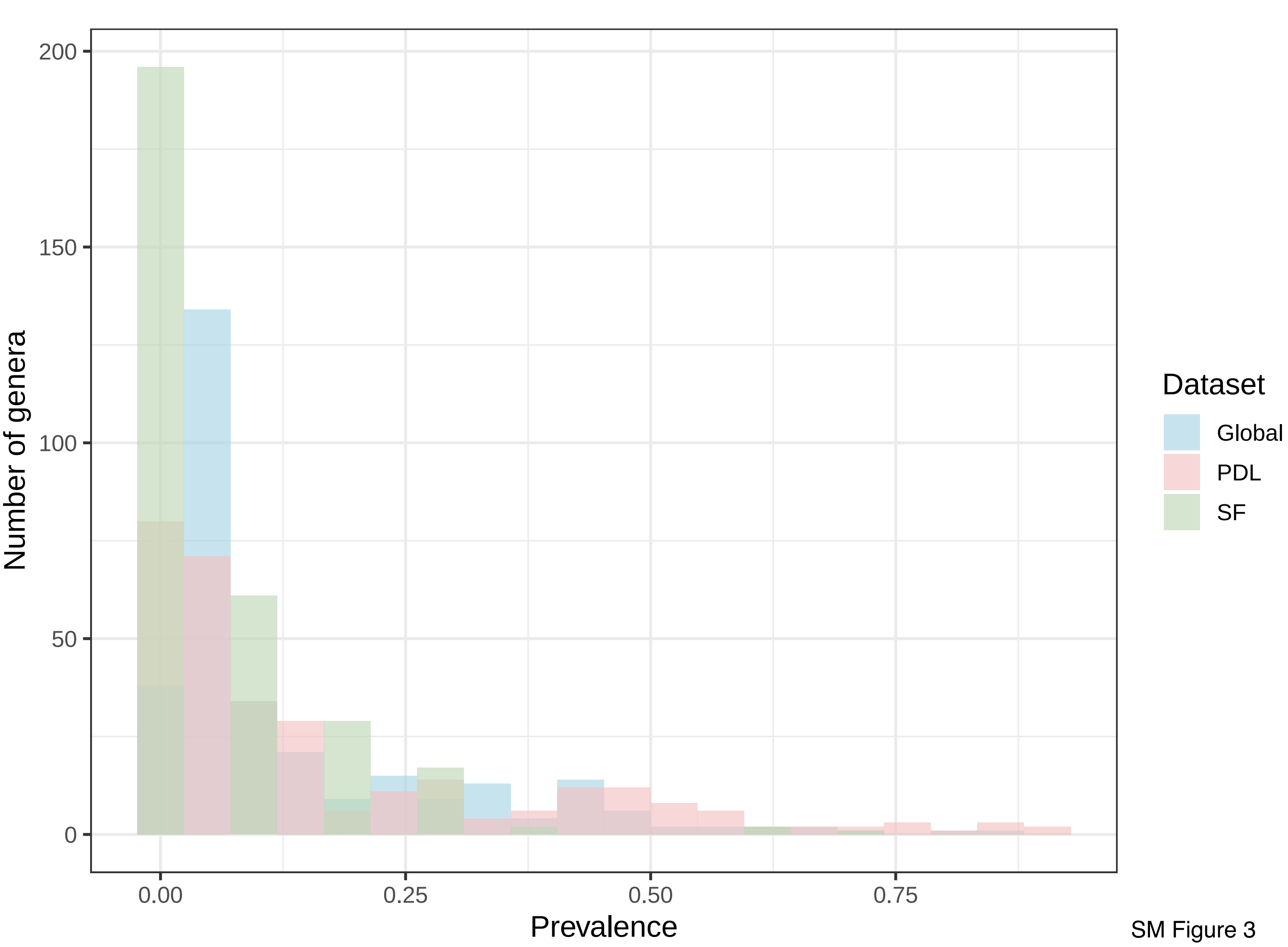
Number of genera
200
150
100
50
0
0.00
0.25
0.50
0.75
Prevalence
Dataset
Global
PDL
SF

SM Figure 3
