# Supplementary material for: Hydrogeological compartmentalization outweighs hydrochemistry in structuring microbial communities across contrasting subsurface habitats
Source: Front Microbiol. 2026 Sep 28;17:1919949. doi: 10.3389/fmicb.2026.1919949 (PMC13656802; doi:10.3389/fmicb.2026.1919949)
Supplement: Supplementary file 1 [file Data_sheet_1.zip › Supplementary Material/Supplementary_Material.docx]

Supplementary Material

# 1 Supplementary Figures and Tables

## 1.1 Supplementary Figures

**Supplementary Figure 1.** Hierarchical clustering of hydrochemical variables based on pairwise correlations in groundwater samples from (A) Pointe-du-Lac (PDL) and (B) Saint-Flavien (SF). Branch lengths represent correlation-based dissimilarities among hydrochemical variables.

**Supplementary Figure 2.** Relative abundance of *Acetobacterium* ASVs across groundwater samples from Pointe-du-Lac (PDL) and Saint-Flavien (SF). Bars show the contribution of individual ASVs to the total bacterial community within each sample. Less abundant ASVs were grouped as “Other *Acetobacterium* ASVs”.

**Supplementary Figure 3.** Prevalence distribution of bacterial genera in groundwater samples from the complete dataset (Global) and within each groundwater system (PDL and SF). Prevalence was calculated as the proportion of samples in which each genus was detected.

**Supplementary Figure 4.** Relative contribution of Core75, Core50, and non-core bacterial genera to community composition in groundwater samples from Pointe-du-Lac (PDL) and Saint-Flavien (SF). Fractions represent the summed relative abundance of genera within each occupancy category for individual samples.

**Supplementary Figure 5.** Archaeal read counts detected in groundwater samples from Pointe-du-Lac (PDL) and Saint-Flavien (SF). Read counts are displayed on a logarithmic scale. Each point represents an individual groundwater sample.

**Supplementary Figure 6.** Detection frequency of methanogen-associated archaeal lineages across groundwater wells from Pointe-du-Lac (PDL) and Saint-Flavien (SF). Cell shading indicates the proportion of archaeal-positive samples from each well in which a given lineage was detected. White cells indicate no detection.

**Supplementary Figure 7.** Principal coordinates analysis (PCoA) of bacterial community composition based on Bray–Curtis dissimilarities, with significant hydrochemical variables fitted using environmental vector fitting (envfit). Vectors represent hydrochemical variables significantly associated with bacterial community turnover.

## 1.2 Supplementary Tables

**Table 1**. Raw hydrochemical characteristics of groundwater samples from Pointe-du-Lac (PDL) and Saint-Flavien (SF). Cond, conductivity. Anion concentrations: mg/L, elemental concentrations: ppm.

| **Sample_ID** | **pH** | **Cond** | **F^-^** | **Cl^-^** | **NO_2_^-^** | **Br^-^** | **NO_3_^-^** | **PO_4_^3-^** | **SO_4_^2-^** |
| --- | --- | --- | --- | --- | --- | --- | --- | --- | --- |
| PDL25_W6 | 5.95 | 57.77 | NA | NA | NA | NA | NA | NA | 256.5888 |
| PDL25_W6-II | 5.87 | 27.82 | NA | NA | NA | NA | NA | NA | 407.4495 |
| PDL25_W7 | 4.13 | 88.37 | NA | NA | NA | NA | NA | NA | 82.938 |
| PDL25_W7-II | 4.62 | 123.7 | NA | NA | NA | NA | NA | NA | 0.24 |
| PDL25_W1 | 4.8 | 168 | NA | NA | NA | NA | NA | NA | 1.3768 |
| PDL25_W1-II | 4.91 | 39 | NA | NA | NA | NA | NA | NA | 55.3117 |
| PDL25_W2 | 6.06 | 12.82 | 71.1158 | NA | NA | 4.2 | 19.8 | 0.32 | 279.924 |
| PDL25_W2-II | 7.01 | 11.75 | 52.2082 | NA | NA | 21.454 | NA | NA | 3.3593 |
| PDL25_W8 | 6.27 | 25.78 | 45.8987 | NA | NA | NA | 53.455 | NA | 1.8084 |
| PDL25_W8-II | 6.26 | 31.51 | 2.3438 | NA | NA | NA | 66.7985 | NA | 4.8226 |
| PDL25_W9 | 5.18 | 176.6 | NA | NA | NA | NA | NA | NA | 87.4427 |
| PDL25_W9-II | 5.13 | 172.2 | NA | NA | NA | NA | NA | NA | 120.2054 |
| PDL25_W3 | 6.75 | 23.88 | NA | NA | NA | NA | 55.9334 | NA | 2.8311 |
| PDL25_W3-II | 6.81 | 40.28 | NA | NA | NA | NA | 62.9001 | NA | 17.2398 |
| PDL25_W4 | 6.85 | 20.35 | 2.0028 | NA | NA | 2.0832 | 33.0211 | NA | 1.0693 |
| PDL25_W4-II | 6.35 | 27.99 | 1.9742 | NA | NA | 0.1796 | 46.332 | NA | 4.051 |
| PDL25_W10-II | 4.95 | 184.4 | NA | NA | NA | NA | NA | NA | 41.6642 |
| PDL25_W11 | 4.57 | 36.93 | 8.4021 | NA | NA | NA | NA | 0.32 | 28.6827 |
| PDL25_W11-II | 6.92 | 23.4 | NA | NA | NA | 2.6202 | 32.2748 | NA | 1.7569 |
| SF25_W1 | 6.2 | 209 | 2.5531 | 118.626 | 1.0284 | 1.0072 | NA | 0.32 | 12.5698 |
| SF25_W1-hII | 5.84 | 53.17 | 1.7051 | 3.8802 | 0.8284 | 0.9498 | NA | 0.783 | 10.9523 |
| SF25_W2 | 6.66 | 4.25 | NA | NA | NA | NA | NA | NA | NA |
| SF25_W2-hII | 4.7 | 22.64 | 17.3228 | NA | NA | 4.3064 | 111.2778 | NA | 159.9902 |
| SF25_W3 | 4.66 | 2.551 | 5.749 | 508.2907 | 3.7366 | 9.513 | NA | NA | 73.1478 |
| SF25_W3-hII | 4.81 | 242.6 | 4.2737 | 80.263 | 1.8174 | 1.5323 | NA | NA | 21.9467 |
| SF25_W4 | 5.13 | 84.21 | 0.3604 | 6.1983 | 0.6868 | 0.3196 | NA | NA | 9.3614 |
| SF25_W4-hII | 5.05 | 49.68 | 0.7666 | 20.9364 | 0.7812 | 0.8454 | NA | 0.32 | 9.0794 |
| SF25_W5 | 4.63 | 122.4 | 1.3852 | 2.4693 | 1.1367 | 0.5751 | NA | NA | 11.3213 |
| SF25_W5-hII | 6.54 | 311 | 1.387 | 1.1644 | 1.3089 | 1.1409 | NA | NA | 50.506 |
| SF25_W6 | 6.24 | 73.58 | 2.9379 | 3.74 | NA | NA | NA | NA | 12.3294 |
| SF25_W6-hII | 4.7 | 619 | 0.1563 | 240.5964 | 0.3584 | 0.1855 | NA | NA | 10.1856 |

| **Sample_ID** | **Al** | **Be** | **Ca** | **Co** | **Cu** | **Fe** | **K** |
| --- | --- | --- | --- | --- | --- | --- | --- |
| PDL25_W6 | 0.14 | 0 | 1292.54 | 0 | 0.11 | 684.91 | 605.13 |
| PDL25_W6-II | 0.1 | 0 | 534.96 | 0.01 | 0.03 | 228.19 | 242.55 |
| PDL25_W7 | 1.51 | 0 | 2080.14 | 0.05 | 0.67 | 2454.71 | 2370.39 |
| PDL25_W7-II | 2.3 | 0 | 2467.85 | 0.06 | 1.03 | 2691.4 | 4248.4 |
| PDL25_W1 | 2.68 | 0 | 2142.8 | 0.04 | 0.98 | 2889.89 | 3845.38 |
| PDL25_W1-II | 1.09 | 0 | 997.64 | 0 | 0.1 | 596.79 | 349.85 |
| PDL25_W2 | 0.12 | 0 | 327.95 | 0.01 | 0.11 | 790.62 | 96.3 |
| PDL25_W2-II | 0.31 | 0 | 292.75 | 0.01 | 0.03 | 228.17 | 88.53 |
| PDL25_W8 | 0.18 | 0 | 685.67 | 0.01 | 0.01 | 79.61 | 141.94 |
| PDL25_W8-II | 0.54 | 0 | 764.45 | 0.02 | 0.03 | 185.88 | 177.22 |
| PDL25_W9 | 0.39 | 0 | 2249.81 | 0.11 | 3.33 | 2801.58 | 1541.18 |
| PDL25_W9-II | 0.17 | 0 | 2597.92 | 0 | 0.44 | 1521.17 | 1204.14 |
| PDL25_W3 | 0.14 | 0 | 694.65 | 0 | 0.02 | 188.18 | 142.4 |
| PDL25_W3-II | 0.23 | 0 | 929.62 | 0.01 | 0.14 | 967.9 | 257.83 |
| PDL25_W4 | 0.11 | 0 | 369.24 | 0 | 0.04 | 53.28 | 88.82 |
| PDL25_W4-II | 0.16 | 0 | 680.03 | 0 | 0.03 | 41.47 | 124.81 |
| PDL25_W10-II | 0.12 | 0 | 2416.67 | 0 | 0.01 | 19.12 | 837.69 |
| PDL25_W11 | 0.15 | 0 | 879.02 | 0.01 | 0.12 | 813.21 | 222.11 |
| PDL25_W11-II | 0.09 | 0 | 371.45 | 0 | 0.01 | 10.68 | 98.67 |
| SF25_W1 | 5.22 | 0.57 | 199.57 | 0.97 | 0.74 | 37.39 | 5.5 |
| SF25_W1-hII | 5.31 | 0.55 | 44.6 | 0.39 | 0.7 | 42.32 | 0 |
| SF25_W2 | 4.59 | 0.52 | 6198 | 0.07 | 1.02 | 1748.88 | 132.24 |
| SF25_W2-hII | 7.44 | 0.5 | 26426.69 | 0.37 | 0.92 | 1633.42 | 731.38 |
| SF25_W3 | 4.26 | 0.51 | 1019.07 | 0 | 2.05 | 1073.41 | 27.76 |
| SF25_W3-hII | 4.58 | 0.52 | 1032.35 | 0.01 | 1.48 | 574.28 | 28.55 |
| SF25_W4 | 5.1 | 0.52 | 223.24 | 0 | 1.65 | 139.43 | 4.31 |
| SF25_W4-hII | 3.35 | 0.5 | 36.02 | 0 | 1.19 | 60.21 | 0 |
| SF25_W5 | 3.66 | 0.5 | 40.64 | 0.25 | 1.3 | 763.72 | 1.96 |
| SF25_W5-hII | 3.79 | 0.52 | 649.66 | 0.38 | 1.82 | 503.58 | 0 |
| SF25_W6 | 4.39 | 0.52 | 38.01 | 0.28 | 0.64 | 128.34 | 0 |
| SF25_W6-hII | 3.15 | 0.51 | 491.87 | 0.29 | 0.99 | 618.41 | 10.32 |

| **Sample_ID** | **Mg** | **Mn** | **Na** | **Ni** | **Sb** | **V** | **Zn** |
| --- | --- | --- | --- | --- | --- | --- | --- |
| PDL25_W6 | 1195.22 | 44.86 | 3338.51 | 0.43 | 0.08 | 7.6 | 0.13 |
| PDL25_W6-II | 956.75 | 6.05 | 3046.9 | 0 | 0 | 2.67 | 0.04 |
| PDL25_W7 | 1121.53 | 146.92 | 3713.36 | 1.87 | 0.06 | 29.8 | 5.57 |
| PDL25_W7-II | 1003.23 | 206.43 | 3628.02 | 2.58 | 0.08 | 49.26 | 8.49 |
| PDL25_W1 | 1039.84 | 234.79 | 3257.5 | 2.95 | 0.03 | 48.4 | 11.39 |
| PDL25_W1-II | 1092.31 | 16.58 | 2655.22 | 0.16 | 0 | 4.32 | 0.66 |
| PDL25_W2 | 621.73 | 15.61 | 2215.31 | 0 | 0.02 | 1.1 | 0.04 |
| PDL25_W2-II | 590.68 | 4.92 | 2094.66 | 0.08 | 0.02 | 1 | 0.04 |
| PDL25_W8 | 960.46 | 7.45 | 2284.29 | 0.5 | 0 | 2.94 | 0.04 |
| PDL25_W8-II | 1017.3 | 6.73 | 2274.38 | 0.48 | 0.01 | 3.64 | 0.06 |
| PDL25_W9 | 1041.07 | 231.78 | 2935.35 | 0.69 | 0.06 | 32.58 | 3.56 |
| PDL25_W9-II | 1104.31 | 147.69 | 2689.3 | 0.12 | 0.02 | 24.65 | 0.7 |
| PDL25_W3 | 896.78 | 11.28 | 2171.66 | 0.05 | 0.03 | 2.4 | 0.07 |
| PDL25_W3-II | 949.91 | 31.03 | 2215.74 | 0.09 | 0.02 | 3.66 | 0.07 |
| PDL25_W4 | 933.91 | 1.7 | 2130.54 | 0.03 | 0.01 | 2.4 | 0.14 |
| PDL25_W4-II | 1082.39 | 15.9 | 2117.2 | 0.01 | 0 | 4.39 | 0.76 |
| PDL25_W10-II | 1206.98 | 23.24 | 2155.16 | 0.44 | 0.01 | 9.46 | 0.12 |
| PDL25_W11 | 1074.46 | 16.99 | 2273.12 | 0.11 | 0.03 | 4.43 | 0.23 |
| PDL25_W11-II | 950.3 | 1.36 | 2139.67 | 0.03 | 0 | 2.51 | 0.21 |
| SF25_W1 | 52.42 | 2.71 | 400.39 | 1.42 | 3.13 | 2.85 | 7.83 |
| SF25_W1-hII | 0 | 4.11 | 198.36 | 0 | 2.88 | 2.56 | 14.82 |
| SF25_W2 | 2147.19 | 36.52 | 6180.67 | 2 | 0.1 | 5.53 | 5.34 |
| SF25_W2-hII | 10118.48 | 64.38 | 31161.32 | 1.74 | 2.73 | 17.64 | 5.51 |
| SF25_W3 | 344.5 | 23.84 | 1197.31 | 2.01 | 2.61 | 2.98 | 5.86 |
| SF25_W3-hII | 335.77 | 20.01 | 1215.71 | 1.9 | 2.01 | 2.99 | 4.23 |
| SF25_W4 | 49.86 | 3.98 | 349.31 | 2.41 | 1.39 | 2.72 | 4.97 |
| SF25_W4-hII | 1.63 | 2.53 | 215.52 | 1.35 | 0.99 | 2.87 | 3.54 |
| SF25_W5 | 10.36 | 35.41 | 178.14 | 1.61 | 1.16 | 2.69 | 17.77 |
| SF25_W5-hII | 457.16 | 27.22 | 200.68 | 3.89 | 0 | 2.66 | 10.76 |
| SF25_W6 | 0 | 6.08 | 180.57 | 0.8 | 3.03 | 2.53 | 3.42 |
| SF25_W6-hII | 234.11 | 30.03 | 243.28 | 4.22 | 0.95 | 2.91 | 18.56 |

**Table 2.** Principal component loadings for the hydrochemical variables included in the principal component analysis (PCA).

| **Variable** | **PC1** | **PC2** |
| --- | --- | --- |
| Na  K  Mg  Al  Sb  Zn  Co  Ca  Cu  Ni  F-  cond  V  Mn  Fe  pH  SO42- | -0.35014  -0.34521  -0.34049  0.322683  0.31521  0.3105  0.266933  -0.26146  0.240093  0.214723  -0.17668  0.161747  -0.11632  -0.11071  -0.08394  -0.07016  -0.06245 | -0.1331  -0.19896  -0.13971  -0.17771  -0.03072  -0.25601  -0.05326  -0.27869  -0.28314  -0.30022  0.085231  -0.17992  -0.36547  -0.39851  -0.35569  0.318524  -0.11972 |

**Table 3**. Univariate comparisons of hydrochemical variables between Pointe-du-Lac (PDL) and Saint-Flavien (SF), using Wilcoxon rank-sum tests. p_adj_BH, Benjamini–Hochberg-adjusted p-values; Cond, conductivity.

| **Variable** | **median_PDL** | **median_SF** | **p_value** | **p_adj_BH** |
| --- | --- | --- | --- | --- |
| Al | 0.17 | 4.49 | 0.00 | 0.00 |
| Be | 0.00 | 0.52 | 0.00 | 0.00 |
| Br^-^ | 2.62 | 0.98 | 0.30 | 0.37 |
| Ca | 879.02 | 357.56 | 0.08 | 0.14 |
| Co | 0.01 | 0.27 | 0.01 | 0.03 |
| Cond | 36.93 | 78.90 | 0.25 | 0.35 |
| Cu | 0.10 | 1.11 | 0.00 | 0.00 |
| F^-^ | 8.40 | 1.71 | 0.05 | 0.08 |
| Fe | 596.79 | 538.93 | 0.64 | 0.70 |
| K | 242.55 | 4.91 | 0.00 | 0.00 |
| Mg | 1017.30 | 143.27 | 0.00 | 0.01 |
| Mn | 16.58 | 21.93 | 0.56 | 0.65 |
| Na | 2274.38 | 296.30 | 0.00 | 0.01 |
| Ni | 0.12 | 1.82 | 0.00 | 0.01 |
| pH | 5.95 | 5.09 | 0.27 | 0.36 |
| PO_4_^3-^ | 0.32 | 0.32 | 0.68 | 0.70 |
| Sb | 0.02 | 1.70 | 0.00 | 0.00 |
| SO_4_^2-^ | 17.24 | 12.33 | 0.70 | 0.70 |
| V | 4.32 | 2.86 | 0.25 | 0.35 |
| Zn | 0.14 | 5.69 | 0.00 | 0.00 |

**Table 4**. Single-factor PERMANOVA analyses of bacterial community composition.

**Site variable**

| **Term** | **Df** | **SumOfSqs** | **R2** | **F** | **Pr(>F)** |
| --- | --- | --- | --- | --- | --- |
| Site | 1 | 0.438043 | 0.040508 | 1.266533 | 0.196 |
| Residual | 30 | 10.37579 | 0.959492 |  |  |
| Total | 31 | 10.81384 | 1 |  |  |

**Well variable**

| **Term** | **Df** | **SumOfSqs** | **R2** | **F** | **Pr(>F)** |
| --- | --- | --- | --- | --- | --- |
| Well | 16 | 7.066875 | 0.653503 | 1.768151 | 0.002 |
| Residual | 15 | 3.746962 | 0.346497 |  |  |
| Total | 31 | 10.81384 | 1 |  |  |

**Year variable**

| **Term** | **Df** | **SumOfSqs** | **R2** | **F** | **Pr(>F)** |
| --- | --- | --- | --- | --- | --- |
| Sampling_year | 1 | 0.724079 | 0.066959 | 2.152914 | 0.03 |
| Residual | 30 | 10.08976 | 0.933041 |  |  |
| Total | 31 | 10.81384 | 1 |  |  |

**Table 5**. Marginal PERMANOVA analyses of bacterial community composition.

| **Term** | **Df** | **SumOfSqs** | **R2** | **F** | **Pr(>F)** |
| --- | --- | --- | --- | --- | --- |
| Site | 0 | -1.8E-15 | -1.6E-16 | -Inf |  |
| Well | 15 | 6.368116 | 0.588886 | 1.806253 | 0.001 |
| Sampling_year | 1 | 0.456406 | 0.042206 | 1.941825 | 0.05 |
| Residual | 14 | 3.290556 | 0.304291 |  |  |
| Total | 31 | 10.81384 | 1 |  |  |

**Table 6**. Within-site PERMANOVA analyses of bacterial community composition.

| **Site_subset** | **Term** | **Df** | **SumOfSqs** | **R2** | **F** | **Pr(>F)** |
| --- | --- | --- | --- | --- | --- | --- |
| Pointe-du-Lac | Well | 10 | 4.979234 | 0.7566 | 3.550293 | 0.001 |
| Pointe-du-Lac | Sampling_year | 1 | 0.339596 | 0.051602 | 2.421385 | 0.02 |
| Pointe-du-Lac | Residual | 9 | 1.262237 | 0.191798 |  |  |
| Pointe-du-Lac | Total | 20 | 6.581066 | 1 |  |  |

| **Site_subset** | **Term** | **Df** | **SumOfSqs** | **R2** | **F** | **Pr(>F)** |
| --- | --- | --- | --- | --- | --- | --- |
| Saint-Flavien | Well | 5 | 1.649598 | 0.434708 | 1.097576 | 0.362 |
| Saint-Flavien | Sampling_year | 1 | 0.942772 | 0.248443 | 3.136411 | 0.005 |
| Saint-Flavien | Residual | 4 | 1.202357 | 0.316849 |  |  |
| Saint-Flavien | Total | 10 | 3.794727 | 1 |  |  |

**Table 7.** BLAST identification of the dominant *Acetobacterium* amplicon sequence variants (ASVs).

| **ASV** | **Closest match** | **Identity (%)** | **Query coverage (%)** |
| --- | --- | --- | --- |
| ASV1 | *Acetobacterium carbinolicum* 9FOS | 100 | 100 |
|  | *Acetobacterium* sp. KB-1 | 100 | 100 |
|  | Uncultured *Acetobacterium* sp. T1-3-4 | 100 | 100 |
|  | *Acetobacterium carbinolicum* 16427 | 100 | 100 |
| ASV4 | *Acetobacterium malicum* 11527 | 100 | 100 |
|  | *Acetobacterium wieringae* CH1 | 100 | 100 |
|  | Uncultured *Acetobacterium* sp. MBR | 100 | 100 |
|  | *Acetobacterium* sp. AP-FeEnrich9 | 100 | 100 |

**Table 8**. Distance-based redundancy analysis (db-RDA) of bacterial community composition constrained by hydrochemical gradients.

| **Term** | **Df** | **SumOfSqs** | **F** | **Pr(>F)** |
| --- | --- | --- | --- | --- |
| ChemPC1 | 1 | 0.548484 | 1.893683 | 0.031 |
| ChemPC2 | 1 | 1.172833 | 4.049292 | 0.001 |
| Residual | 17 | 4.923862 |  |  |

**Table 9**. Spearman correlations between dominant bacterial genera and hydrochemical gradients at global and site-specific scales.

**Global**

| **Genus** | **Axis** | **rho** | **p_value** | **p_BH** |
| --- | --- | --- | --- | --- |
| Acetobacterium | ChemPC1 | -0.31325 | 0.178674 | 0.310737 |
| Allorhizobium-Neorhizobium-Pararhizobium-Rhizobium | ChemPC1 | 0.21988 | 0.351602 | 0.498473 |
| Desulfosporosinus | ChemPC1 | 0.17482 | 0.46101 | 0.614681 |
| Desulfovibrio | ChemPC1 | 0.08355 | 0.726189 | 0.794509 |
| Hoeflea | ChemPC1 | 0.219798 | 0.351786 | 0.498473 |
| Hydrogenophaga | ChemPC1 | 0.411321 | 0.071583 | 0.230147 |
| Polaromonas | ChemPC1 | 0.645288 | 0.002123 | 0.016983 |
| Proteiniclasticum | ChemPC1 | 0.35811 | 0.121062 | 0.254866 |
| Proteiniphilum | ChemPC1 | 0.080788 | 0.734921 | 0.794509 |
| Rhodococcus | ChemPC1 | 0.125784 | 0.597215 | 0.702606 |
| Rhodoferax | ChemPC1 | 0.370492 | 0.107827 | 0.254027 |
| Sulfuricurvum | ChemPC1 | 0.215555 | 0.361393 | 0.498473 |
| Sulfurimonas | ChemPC1 | 0.566146 | 0.009263 | 0.05293 |
| Sulfurospirillum | ChemPC1 | 0.284081 | 0.224806 | 0.359689 |
| Thiobacillus | ChemPC1 | 0.047276 | 0.843107 | 0.887481 |
| Trichococcus | ChemPC1 | 0.370361 | 0.107962 | 0.254027 |
| unclassified_Arcobacteraceae | ChemPC1 | -0.11408 | 0.632018 | 0.722306 |
| unclassified_Methylophagaceae | ChemPC1 | 0.029702 | 0.901072 | 0.924177 |
| unclassified_TRA3-20 | ChemPC1 | -0.5181 | 0.019278 | 0.08568 |
| Nocardioides | ChemPC1 | 0.000927 | 0.996905 | 0.996905 |
| Acetobacterium | ChemPC2 | 0.668675 | 0.001267 | 0.012672 |
| Allorhizobium-Neorhizobium-Pararhizobium-Rhizobium | ChemPC2 | -0.29669 | 0.204004 | 0.340007 |
| Desulfosporosinus | ChemPC2 | 0.555011 | 0.011082 | 0.055409 |
| Desulfovibrio | ChemPC2 | 0.670807 | 0.001206 | 0.012672 |
| Hoeflea | ChemPC2 | 0.401407 | 0.079396 | 0.230147 |
| Hydrogenophaga | ChemPC2 | 0.245829 | 0.296147 | 0.45561 |
| Nocardioides | ChemPC2 | 0.14739 | 0.535188 | 0.685435 |
| Polaromonas | ChemPC2 | -0.4 | 0.080552 | 0.230147 |
| Proteiniclasticum | ChemPC2 | 0.41637 | 0.067832 | 0.230147 |
| Proteiniphilum | ChemPC2 | 0.826527 | 7.07E-06 | 0.000283 |
| Rhodococcus | ChemPC2 | 0.142717 | 0.548348 | 0.685435 |
| Rhodoferax | ChemPC2 | -0.13085 | 0.582393 | 0.702606 |
| Sulfuricurvum | ChemPC2 | 0.384434 | 0.094215 | 0.25124 |
| Sulfurimonas | ChemPC2 | 0.323076 | 0.16471 | 0.30438 |
| Sulfurospirillum | ChemPC2 | 0.321135 | 0.167409 | 0.30438 |
| Thiobacillus | ChemPC2 | 0.571019 | 0.008547 | 0.05293 |
| Trichococcus | ChemPC2 | 0.362131 | 0.116643 | 0.254866 |
| unclassified_Arcobacteraceae | ChemPC2 | 0.416141 | 0.067999 | 0.230147 |
| unclassified_Methylophagaceae | ChemPC2 | 0.705221 | 0.000515 | 0.010294 |
| unclassified_TRA3-20 | ChemPC2 | -0.33915 | 0.143517 | 0.287034 |

**Within site PDL**

| **Genus** | **Axis** | **rho** | **p_value** | **p_BH** |
| --- | --- | --- | --- | --- |
| Acetobacterium | ChemPC1 | -0.7471 | 0.00137 | 0.017356 |
| Allorhizobium-Neorhizobium-Pararhizobium-Rhizobium | ChemPC1 | 0.375 | 0.168433 | 0.35558 |
| Desulfosporosinus | ChemPC1 | -0.3087 | 0.262934 | 0.499575 |
| Desulfovibrio | ChemPC1 | -0.57464 | 0.025048 | 0.118978 |
| Hoeflea | ChemPC1 | -0.19952 | 0.475886 | 0.821985 |
| Hydrogenophaga | ChemPC1 | 0.076376 | 0.786748 | 0.918743 |
| Nocardioides | ChemPC1 | -0.1382 | 0.623289 | 0.918743 |
| Polaromonas | ChemPC1 | 0.785714 | 0.000516 | 0.017239 |
| Proteiniclasticum | ChemPC1 | -0.16846 | 0.548403 | 0.868305 |
| Proteiniphilum | ChemPC1 | -0.7276 | 0.002108 | 0.020027 |
| Rhodococcus | ChemPC1 | -0.07914 | 0.779206 | 0.918743 |
| Rhodoferax | ChemPC1 | 0.356423 | 0.192236 | 0.384472 |
| Sulfuricurvum | ChemPC1 | -0.1807 | 0.519265 | 0.857916 |
| Sulfurimonas | ChemPC1 | 0.121429 | 0.666401 | 0.918743 |
| Sulfurospirillum | ChemPC1 | -0.05423 | 0.847774 | 0.918743 |
| Thiobacillus | ChemPC1 | -0.39082 | 0.14976 | 0.334759 |
| Trichococcus | ChemPC1 | 0.027664 | 0.922039 | 0.922039 |
| unclassified_Arcobacteraceae | ChemPC1 | -0.53115 | 0.041608 | 0.158112 |
| unclassified_Methylophagaceae | ChemPC1 | -0.54587 | 0.035292 | 0.149009 |
| Acetobacterium | ChemPC2 | 0.673816 | 0.005882 | 0.039592 |
| Allorhizobium-Neorhizobium-Pararhizobium-Rhizobium | ChemPC2 | -0.47857 | 0.071131 | 0.219522 |
| Desulfosporosinus | ChemPC2 | 0.402814 | 0.136574 | 0.324363 |
| Desulfovibrio | ChemPC2 | 0.472805 | 0.0751 | 0.219522 |
| Hoeflea | ChemPC2 | 0.199525 | 0.475886 | 0.821985 |
| Hydrogenophaga | ChemPC2 | -0.11638 | 0.679568 | 0.918743 |
| Nocardioides | ChemPC2 | 0.072291 | 0.79793 | 0.918743 |
| Polaromonas | ChemPC2 | -0.76429 | 0.000907 | 0.017239 |
| Proteiniclasticum | ChemPC2 | 0.064517 | 0.819311 | 0.918743 |
| Proteiniphilum | ChemPC2 | 0.670255 | 0.006251 | 0.039592 |
| Rhodococcus | ChemPC2 | -0.06893 | 0.807164 | 0.918743 |
| Rhodoferax | ChemPC2 | -0.42916 | 0.110415 | 0.279719 |
| Sulfuricurvum | ChemPC2 | 0.052705 | 0.852018 | 0.918743 |
| Sulfurimonas | ChemPC2 | -0.075 | 0.790511 | 0.918743 |
| Sulfurospirillum | ChemPC2 | 0.036801 | 0.896403 | 0.92063 |
| Thiobacillus | ChemPC2 | 0.495581 | 0.060303 | 0.208321 |
| Trichococcus | ChemPC2 | -0.04611 | 0.870389 | 0.918743 |
| unclassified_Arcobacteraceae | ChemPC2 | 0.433405 | 0.106554 | 0.279719 |
| unclassified_Methylophagaceae | ChemPC2 | 0.651279 | 0.008537 | 0.046343 |

**Within site SF**

| **Genus** | **Axis** | **rho** | **p_value** | **p_BH** |
| --- | --- | --- | --- | --- |
| Acetobacterium | ChemPC1 | 0.5 | 0.391002 | 0.934713 |
| Allorhizobium-Neorhizobium-Pararhizobium-Rhizobium | ChemPC1 | -0.2052 | 0.740582 | 0.934713 |
| Polaromonas | ChemPC1 | -0.1118 | 0.857945 | 0.934713 |
| unclassified_TRA3-20 | ChemPC1 | -0.78262 | 0.117614 | 0.934713 |
| Acetobacterium | ChemPC2 | 0.4 | 0.504632 | 0.934713 |
| Allorhizobium-Neorhizobium-Pararhizobium-Rhizobium | ChemPC2 | -0.0513 | 0.934713 | 0.934713 |
| Polaromonas | ChemPC2 | -0.44721 | 0.450185 | 0.934713 |
| unclassified_TRA3-20 | ChemPC2 | -0.1118 | 0.857945 | 0.934713 |

**Table 10**. Spearman correlations between bacterial genera and methanogen-associated archaeal lineages. Only data with significant p-values are shown.

| **bacterial_taxon** | **methanogen_taxon** | **rho** | **p_adj_BH_global** |
| --- | --- | --- | --- |
| Anaerovorax | Methanolobus | 0.706147 | 0.029602 |
| Anaerovorax | Unclassified_Methanobacteriaceae | 0.762261 | 0.010517 |
| Azoarcus | Methanocorpusculum | 0.826688 | 0.00341 |
| Candidatus Deferrimonas | Unclassified_Methanosarciniales | 0.709511 | 0.029602 |
| Demequina | Unclassified_Methanosarciniales | 0.735346 | 0.01789 |
| Desulfocarbo | Methanolobus | 0.835436 | 0.003093 |
| Desulfomicrobium | Methanolobus | 0.829952 | 0.003384 |
| Desulfurivibrio | Methanolobus | 0.762448 | 0.010517 |
| Ercella | Methanolobus | 0.688902 | 0.039731 |
| EUB33-2 | Methanolobus | 0.87663 | 0.001609 |
| EUB33-2 | Unclassified_Methanosarcinaceae | 0.702825 | 0.030959 |
| Geothermobacter | Methanocalculus | 0.690337 | 0.039339 |
| Geothermobacter | Methanolobus | 0.700586 | 0.031874 |
| Izimaplasma | Methanolobus | 0.721964 | 0.023223 |
| Proteiniphilum | Unclassified_Methanobacteriaceae | 0.764131 | 0.010517 |
| Rhodococcus | Methanocorpusculum | 0.749838 | 0.013189 |
| Rhodococcus | Unclassified_Methanosarciniales | 0.840596 | 0.002927 |
| Ruminiclostridium | Unclassified_Methanosarcinaceae | 0.681491 | 0.042978 |
| Sideroxydans | Methanocorpusculum | 0.781128 | 0.007962 |
| Sideroxydans | Unclassified_Methanosarciniales | 0.857135 | 0.001609 |
| Smithella | Methanocalculus | 0.811859 | 0.00455 |
| Soehngenia | Methanocalculus | 0.753294 | 0.012795 |
| Syntrophorhabdus | Methanocalculus | 0.86164 | 0.001609 |
| Syntrophorhabdus | Methanosaeta | 0.671671 | 0.049565 |
| Thermovirga | Methanocalculus | 0.769195 | 0.009655 |
| Thermovirga | Methanolobus | 0.686878 | 0.039815 |
| Thermovirga | Unclassified_Methanobacteriaceae | 0.732681 | 0.018639 |
| Trichloromonas | Methanolobus | 0.675299 | 0.047152 |
| unclassified_Anaerovoracaceae | Methanolobus | 0.777292 | 0.008579 |
| unclassified_Bacteroidia | Methanobacterium | 0.70573 | 0.029602 |
| unclassified_Bacteroidia | Unclassified_Methanobacteriaceae | 0.78903 | 0.007238 |
| unclassified_Christensenellaceae | Methanolobus | 0.792742 | 0.007238 |
| unclassified_Christensenellaceae | Unclassified_Methanobacteriaceae | 0.747222 | 0.013746 |
| unclassified_Clostridia UCG-014 | Unclassified_Methanosarciniales | 0.761171 | 0.010517 |
| unclassified_Desulfuromonadaceae | Methanocalculus | 0.741581 | 0.01558 |
| unclassified_Dysgonomonadaceae | Methanobacterium | 0.70573 | 0.029602 |
| unclassified_Dysgonomonadaceae | Methanolobus | 0.722357 | 0.023223 |
| unclassified_Firmicutes | Methanolobus | 0.750366 | 0.013189 |
| unclassified_Gaiellales | Unclassified_Methanosarciniales | 0.813515 | 0.00455 |
| unclassified_Hungateiclostridiaceae | Methanolobus | 0.678095 | 0.045313 |
| unclassified_Hydrogenoanaerobacterium | Unclassified_Methanobacteriaceae | 0.790034 | 0.007238 |
| unclassified_Izemoplasmataceae | Methanolobus | 0.784039 | 0.007626 |
| unclassified_Lachnospirales | Methanocalculus | 0.687408 | 0.039815 |
| unclassified_Lachnospirales | Methanolobus | 0.784041 | 0.007626 |
| unclassified_Lentimicrobiaceae | Unclassified_Methanosarcinaceae | 0.713656 | 0.027774 |
| unclassified_Magnetospiraceae | Methanocalculus | 0.671104 | 0.049565 |
| unclassified_Rikenellaceae | Methanolobus | 0.773484 | 0.009236 |
| unclassified_Spirochaetaceae | Methanolobus | 0.819306 | 0.004128 |
| unclassified_Synergistaceae | Methanobacterium | 0.70573 | 0.029602 |
| unclassified_Synergistaceae | Unclassified_Methanobacteriaceae | 0.862553 | 0.001609 |
| unclassified_Syntrophales | Methanocalculus | 0.793242 | 0.007238 |
| unclassified_Syntrophales | Methanosaeta | 0.769895 | 0.009655 |
| unclassified_UBA12409 | Methanocalculus | 0.681851 | 0.042978 |
| unclassified_UBA12409 | Methanolobus | 0.799993 | 0.006536 |
