# Supplementary material for: Hydrogeological compartmentalization outweighs hydrochemistry in structuring microbial communities across contrasting subsurface habitats
Source: Front Microbiol. 2026 Sep 28;17:1919949. doi: 10.3389/fmicb.2026.1919949 (PMC13656802; doi:10.3389/fmicb.2026.1919949)
Supplement: Supplementary file 1 [file Data_sheet_1.zip › Supplementary Material/Figure S7.pdf]

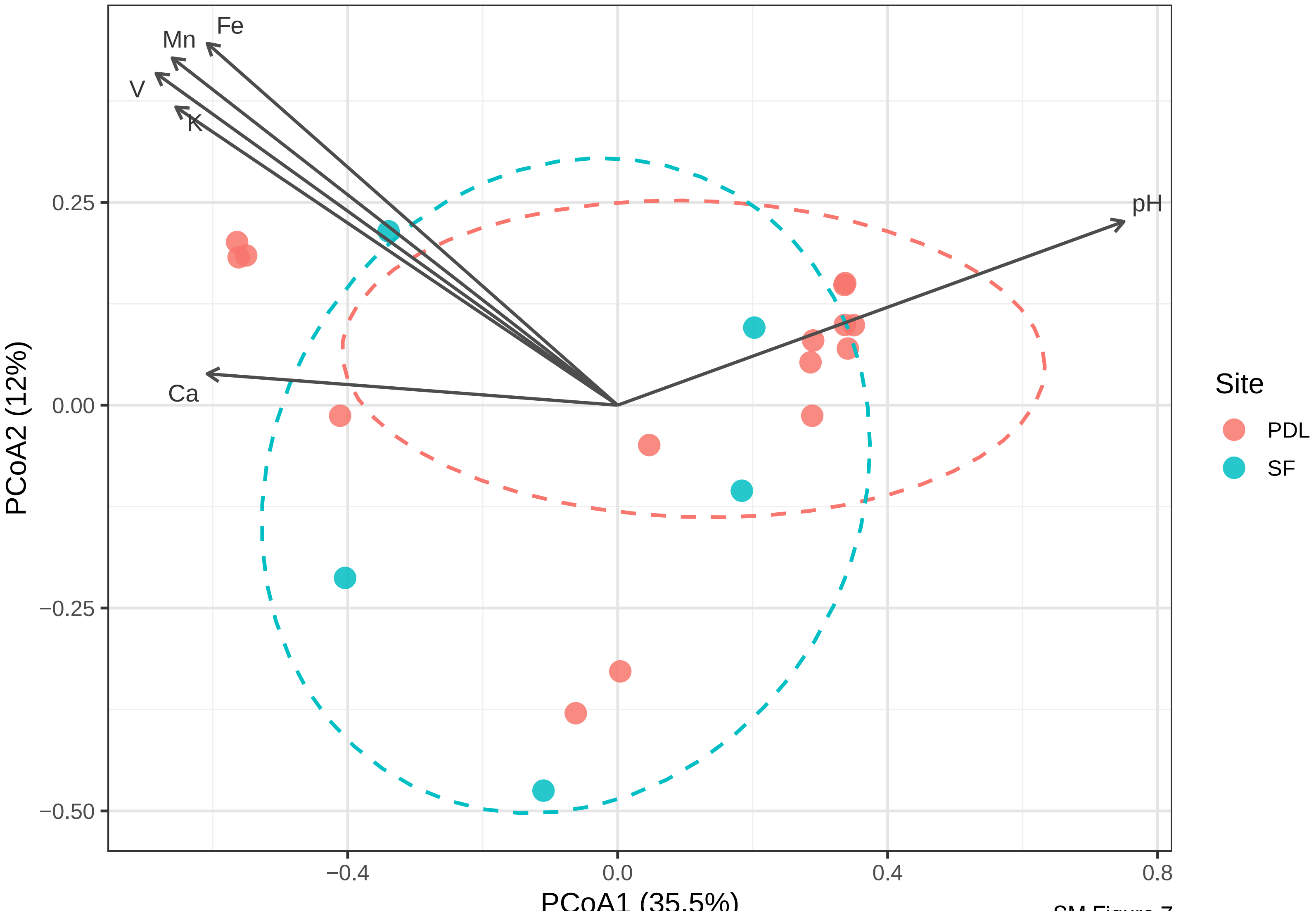
Fe
Mn
V
K
pH
Ca
0.25
0.00
−0.25
−0.50
−0.4
0.0
0.4
0.8
PCoA2 (12%)
PCoA1 (35.5%)
Site
PDL
SF

SM Figure 7
