# Supplementary material for: Hydrogeological compartmentalization outweighs hydrochemistry in structuring microbial communities across contrasting subsurface habitats
Source: Front Microbiol. 2026 Sep 28;17:1919949. doi: 10.3389/fmicb.2026.1919949 (PMC13656802; doi:10.3389/fmicb.2026.1919949)
Supplement: Supplementary file 1 [file Data_sheet_1.zip › Supplementary Material/Figure S6.pdf]

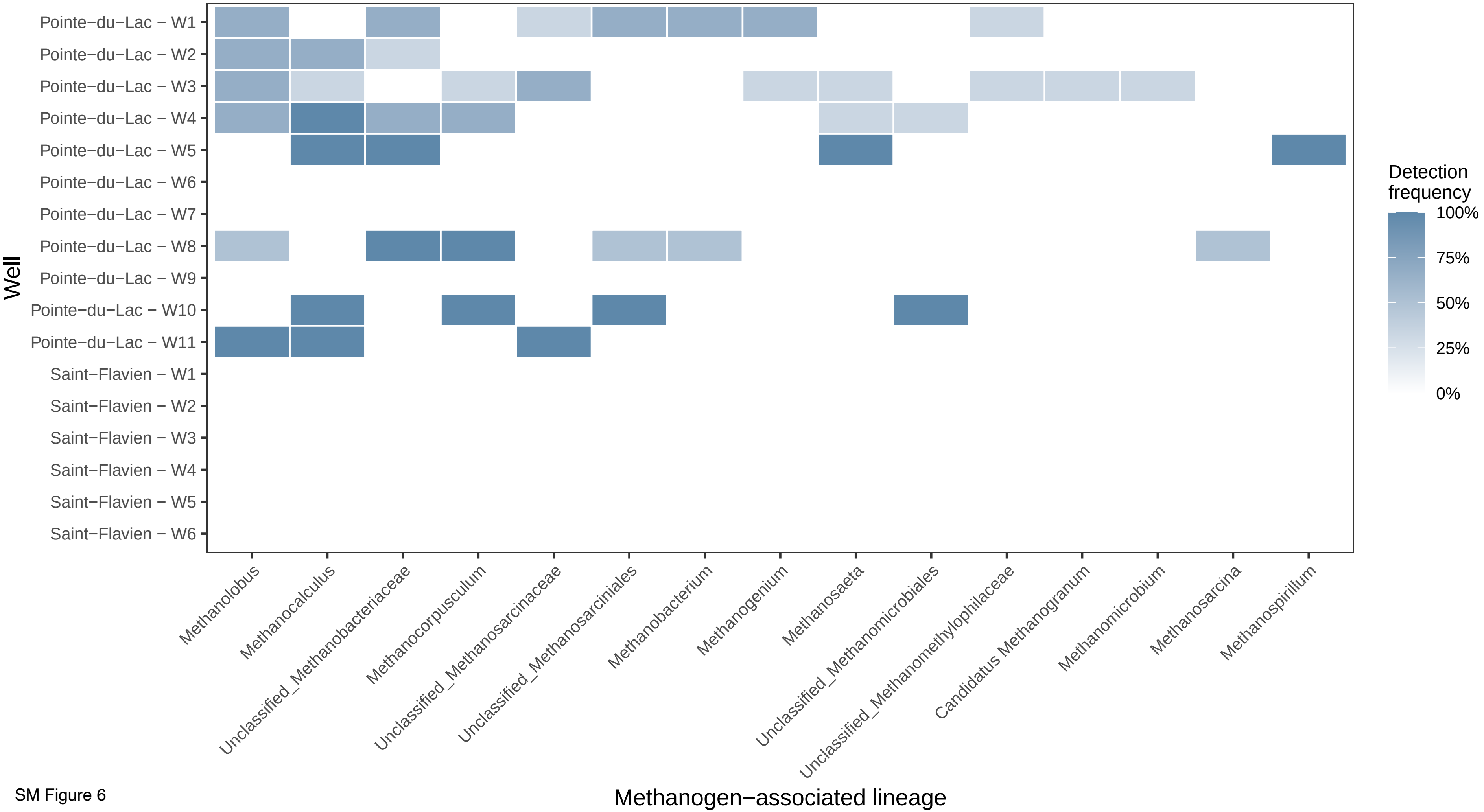

Pointe−du−Lac − W1
Pointe−du−Lac − W2
Pointe−du−Lac − W3
Pointe−du−Lac − W4
Pointe−du−Lac − W5
Pointe−du−Lac − W6
Pointe−du−Lac − W7
Pointe−du−Lac − W8
Pointe−du−Lac − W9
Pointe−du−Lac − W10
Pointe−du−Lac − W11
Saint−Flavien − W1
Saint−Flavien − W2
Saint−Flavien − W3
Saint−Flavien − W4
Saint−Flavien − W5
Saint−Flavien − W6
Well
Methanolobus
Methanocalculus
Unclassified_Methanobacteriaceae
Methanocorpusculum
Unclassified_Methanosarcinaceae
Unclassified_Methanosarciniales
Methanobacterium
Methanogenium
Methanosaeta
Unclassified_Methanomicrobiales
Unclassified_Methanomethylophilaceae
Candidatus Methanogranum
Methanomicrobium
Methanosarcina
Methanospirillum
Methanogen−associated lineage
Detection frequency
100%
75%
50%
25%
0%

SM Figure 6
