# Supplementary material for: Hydrogeological compartmentalization outweighs hydrochemistry in structuring microbial communities across contrasting subsurface habitats
Source: Front Microbiol. 2026 Sep 28;17:1919949. doi: 10.3389/fmicb.2026.1919949 (PMC13656802; doi:10.3389/fmicb.2026.1919949)
Supplement: Supplementary file 1 [file Data_sheet_1.zip › Supplementary Material/Figure S4.pdf]

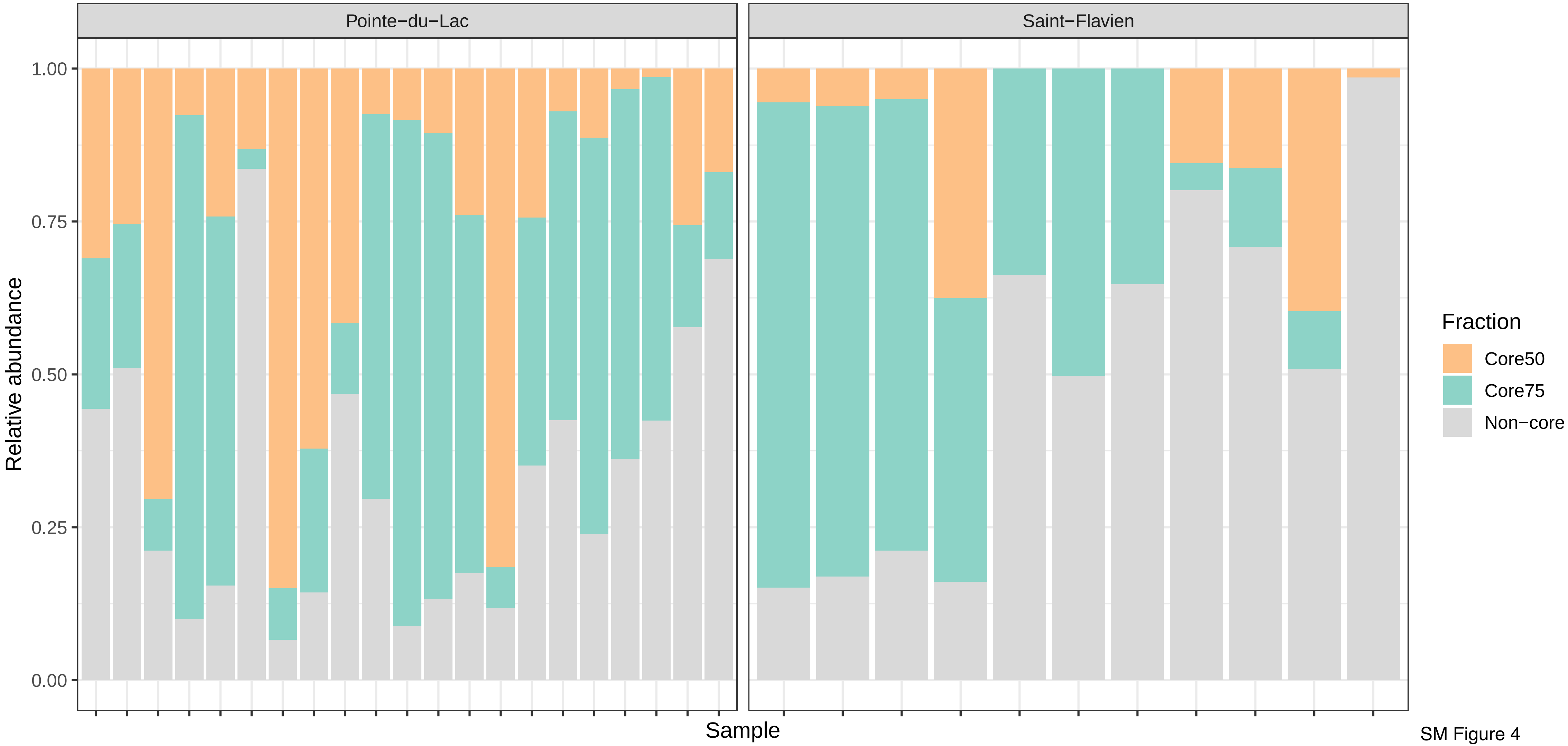

Pointe−du−Lac
Saint−Flavien
1.00
0.75
0.50
0.25
0.00
Relative abundance
Sample
Fraction
Core50
Core75
Non−core

SM Figure 4
