# Supplementary material for: Hydrogeological compartmentalization outweighs hydrochemistry in structuring microbial communities across contrasting subsurface habitats
Source: Front Microbiol. 2026 Sep 28;17:1919949. doi: 10.3389/fmicb.2026.1919949 (PMC13656802; doi:10.3389/fmicb.2026.1919949)
Supplement: Supplementary file 1 [file Data_sheet_1.zip › Supplementary Material/Figure S5.pdf]

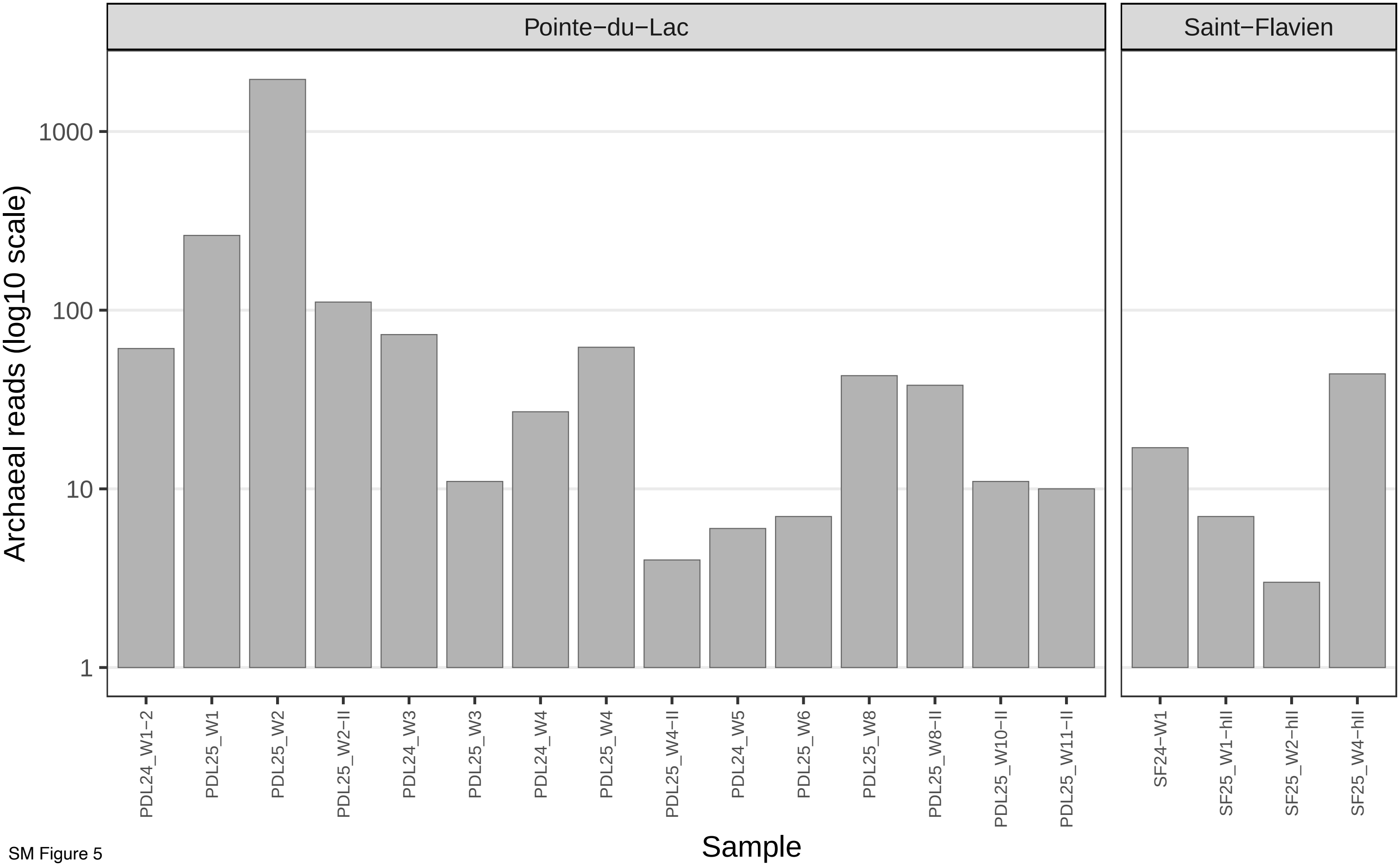
Pointe−du−Lac
Saint−Flavien
Archaeal reads (log10 scale)
1000
100
10
1
Sample
PDL24_W1−2
PDL25_W1
PDL25_W2
PDL25_W2−II
PDL24_W3
PDL25_W3
PDL24_W4
PDL25_W4
PDL25_W4−II
PDL24_W5
PDL25_W6
PDL25_W8
PDL25_W8−II
PDL25_W10−II
PDL25_W11−II
SF24−W1
SF25_W1−hII
SF25_W2−hII
SF25_W4−hII

SM Figure 5
